# Multi-omics analysis reveals LARP1 as a key integrator of translation and metabolism in AML

**DOI:** 10.1038/s41389-026-00623-3

**Published:** 2026-05-16

**Authors:** Dominik A. Nahotko, Brian Lee, Emely Lopez Fajardo, Aneta H. Baran, Szymon K. Filip, Peter A. Faull, Elizabeth T. Bartom, Masha Kocherginsky, Peng Gao, Jason Miska, Diana Saleiro, Elspeth M. Beauchamp, Leonidas C. Platanias

**Affiliations:** 1 Robert H. Lurie Comprehensive Cancer Center, Northwestern University, Chicago, IL USA; 2 Department of Medicine, Jesse Brown Veterans Affairs Medical Center, Chicago, IL USA; 3 Northwestern University Proteomics Core, Proteomics Center of Excellence, Northwestern University, Chicago, IL USA; 4 Division of Biostatistics and Informatics, Department of Preventive Medicine, Feinberg School of Medicine, Northwestern University, Chicago, IL USA; 5 Department of Biochemistry and Molecular Genetics, Feinberg School of Medicine, Northwestern University, Chicago, IL USA; 6 Division of Pulmonary and Critical Care, Department of Medicine, Feinberg School of Medicine, Northwestern University, Chicago, IL USA; 7 Department of Neurological Surgery, Feinberg School of Medicine, Northwestern University, Chicago, IL USA; 8 Lou and Jean Malnati Brain Tumor Institute, Feinberg School of Medicine, Northwestern University, Chicago, IL USA; 9 Division of Hematology/Oncology, Department of Medicine, Feinberg School of Medicine, Northwestern University, Chicago, IL USA

**Keywords:** Acute myeloid leukaemia, Oncogenes, Target identification, Cancer metabolism

## Abstract

La-related protein 1 (LARP1) is an RNA-binding protein and downstream effector of mTOR and CDK9 signaling that regulates translation of mRNAs containing a 5’-terminal oligopyrimidine motif. While elevated LARP1 expression has been linked to poor prognosis in acute myeloid leukemia (AML), its mechanistic role remains unclear. Using CRISPR/Cas9-mediated *LARP1* knockout and multi-omics analyses, we investigated LARP1’s role in AML. *LARP1* loss impaired proliferation, clonogenicity, and tumor growth in xenografts, and enhanced sensitivity of AML cells to 5-azacytidine and cytarabine. Polysome profiling and RNA sequencing revealed that LARP1 modulates a distinct set of transcripts involved in mitochondrial function, amino acid metabolism, and cell cycle regulation, independently of mTOR and CDK9. Proteomics analysis uncovered additional effects of LARP1 loss on immune signaling, lysosomal pathways, and protein stability, including changes not evident at the RNA level. Metabolomic profiling showed reprogramming of arginine/creatine metabolism and depletion of pyrimidine biosynthesis intermediates. Cytidine deaminase, a known resistance factor, was downregulated across omics layers upon *LARP1* loss. These findings define LARP1 as a key integrator of translational regulation and metabolic control in AML, supporting leukemic cell survival and promoting drug resistance. Targeting LARP1 may uncover vulnerabilities in leukemia cells, not addressed by current therapies.

## Introduction

Acute myeloid leukemia (AML) is a hematological malignancy with very high mortality, whose pathogenesis is often linked to a series of genetic mutations and chromosomal abnormalities that disrupt normal hematopoiesis, leading to leukemogenesis [1, 2]. Current treatment strategies, primarily involving intensive chemotherapy regimens [2], yield a 5-year survival rate of approximately 32% [3, 4], underscoring the urgent need for new therapeutic approaches.

The mammalian target of rapamycin (mTOR) pathway integrates pro-mitogenic signals to promote cellular growth and survival and is aberrantly activated in approximately 60% of AML patients, conferring proliferation and survival advantages to the transformed leukemic blasts [5]. Although targeting mTOR for AML therapy has been an area of significant interest, clinical efficacy has been constrained due to the dose-limiting toxicity of the studied small molecule inhibitors and activation of compensatory mechanisms related to the mTOR pathway [6, 7]. An example of such is the “mTOR-like” CDK9 complexes, CTORC, recently described by our group, which act as alternative regulators of rpS6 and La-related protein 1 (LARP1), compensating for mTOR pathway inhibition [8].

LARP1 is an RNA-binding protein which is known to interact with mRNAs containing 5’-terminal oligopyrimidine (TOP) motifs [9, 10]. TOP mRNAs typically encode proteins of the translational machinery, but also proteins related to nutrient metabolism and mitochondrial biogenesis [11]. LARP1 plays dual roles in control of TOP mRNA translation by stabilizing these transcripts [12, 13] and competing with eukaryotic translation initiation factor 4E for binding TOP mRNA cap [10], effectively inhibiting their translation under antimitogenic conditions [13]. On the other hand, in a pro-mitogenic cell environment LARP1 is directly phosphorylated by the mTOR complex 1 (mTORC1), which results in release of 5’-end of the TOP mRNA and facilitates initiation of translation [13]. Therefore, LARP1 acts as a molecular switch controlling translation of TOP mRNAs downstream of mTORC1 depending on cell state [13]. Additionally, a previous study by our group described the existence of a novel CTORC complex composed of RICTOR, mSIN1, mLST8 and CDK9, which phosphorylates LARP1 [8]. Although CDK9 inhibition has been shown to block parts of the translation machinery and decrease translation efficiency in AML cells, specific pools of mRNAs translationally affected by CDK9 inhibition remain to be defined [8].

Recent studies have increasingly highlighted amino acid metabolism as a hallmark of AML [14, 15]. AML blasts often exhibit metabolic auxotrophies, particularly for arginine and glutamine, due to downregulation of key biosynthetic enzymes such as ASS1 and reliance on amino acid transporters SLC1A5 [16], and SLC7A1/A2 [17]. Arginine deprivation strategies using pegylated arginine deiminase (ADI-PEG 20) or recombinant arginases (e.g., BCT-100) have demonstrated cytotoxicity against AML blasts in vitro and in vivo [17, 18]. Amino acid metabolism plays also a crucial role in shaping the immunosuppressive microenvironment of AML, as evidenced by the arginase-mediated suppression of T-cell proliferation and monocyte polarization [19, 20].

Increased expression of LARP1 in tumor cells has been correlated with unfavorable prognosis in multiple types of cancers [21–24]. Recent bioinformatic and experimental studies have further demonstrated that LARP1 expression serves as a prognostic marker in AML, with higher LARP1 levels being associated with poorer patient outcomes [25]. Mitochondrial function [26–28] and amino acid metabolism [15] have been established as potential therapeutic vulnerabilities in AML. Our findings raise the possibility that LARP1 contributes to AML pathogenesis by sustaining these critical metabolic programs. As a downstream effector of both mTORC1 [29, 30] and CTORC2 [8], LARP1 may thus represent a novel, targetable node within these metabolic vulnerabilities. This led us to investigate the molecular mechanisms by which LARP1 contributes to AML leukemogenesis by employing multi-omics approaches, including transcriptomic, translatomic, proteomic, and metabolomic analyses, comprehensively comparing CRISPR/Cas9 *LARP1* knockout (KO) to mTOR and CDK9 kinase inhibition.

## Methods

All materials used in this study are listed in the Key Resources Table provided in Supplementary Information.

### Cell culture

U937 and HEL cells were cultured in RPMI (Gibco) supplemented with 10% FBS (Sigma). OCIAML5 cells were cultured in MEM alpha (Gibco) supplemented with 20% FBS and GM-CSF (PeproTech) at 10 ng/ml. C1498 cells were cultured in DMEM (Gibco) supplemented with 10% FBS, 1 mM sodium pyruvate (Gibco), 1.5 g/l sodium bicarbonate (Gibco). Kasumi1 cells were cultured in RPMI (Gibco) supplemented with 20% FBS (Sigma). KG1 cells were cultured in IMDM (Gibco) supplemented with 20% FBS (Sigma). All cell lines were incubated in 5% CO_2_ air at 37 °C. TC20 Automated Cell Counter was used for routine cell counts and viability testing after staining with Trypan Blue Dye 0.4% (BioRad). All cell lines were authenticated by short tandem repeats profiling (Labcorp) and routinely tested for mycoplasma infection with MycoAlert Mycoplasma detection kit (Lonza).

### AML xenograft in vivo study

All animal studies were approved by the Northwestern University Institutional Animal Care and Use Committee and performed accordingly. Five- to six-week-old Athymic Nude female mice were purchased from Taconic Biosciences. A total of 2.5 × 10^6^ single-guide non-targeting control (sgNT) U937 cells and sg*LARP1* (single-guide *LARP1*-targeting) U937 clone 1 cells were injected subcutaneously in 100 μl DPBS (Gibco) into the right flank of each animal. The sample size for the animal studies was determined based on prior experiments with the same cell line. No randomization method was used. Each experimental group included three to six female mice that were monitored for clinical observations, body weight and tumor growth by caliper measurements, performed without blinding to group allocation, starting on day 9 after injections. Animals were excluded from the analysis if tumors failed to engraft or grow following AML cell line injection by the experimental endpoint. Tumor volume was calculated as previously described [8]. Animals were euthanized when tumor volume exceeded 2000 mm^3^. The study was repeated twice.

### CRISPR/CRISPR-associated protein 9 (Cas9) gene editing

AML cell lines stably expressing Cas9 nuclease and sgRNA targeting *LARP1* (sg*LARP1*) were generated via lentiviral transduction using the Dharmacon Edit-R All-in-one system (Horizon). Control cells carrying a non-targeting sgRNA (sgNT) were created similarly. Additional details are provided in Supplementary Information.

### Immunoblotting

Cells were lysed in Triton-X 100 buffer and immunoblotting was performed as previously described [8]. Chemiluminescent signals were visualized using an ECL substrate and captured on film or digital imaging systems. See Supplementary Information for detailed protocol and antibody information.

### Colony formation assays

Clonogenic assays for sg*LARP1* and sgNT U937, OCIAML5 and C1498-derived leukemic progenitors were assessed using MethoCult Classic (human cells—STEMCELL Technologies) or MethoCult GF (mouse cells—STEMCELL Technologies) cultures, as previously described [31].

### Cell proliferation assays

sg*LARP1 and* sgNT U937, OCIAML5 and C1498 cells were seeded in technical duplicates on day 0 at equal densities. A total of 10 μL samples were removed for Trypan Blue staining followed by live cell quantification at 24, 48 and 72 h after cell seeding, as previously described [32].

### Cell viability assays

sg*LARP1* and sgNT U937 cells were seeded in triplicates on a 96-well plate, 1500 cells per well, and treated with either vehicle control, or increasing concentrations of 5-azacytidine (TargetMol) or cytarabine (TargetMol). Ninety-six hours after treatment a water-soluble tetrazolium 1 salt reagent (WST-1—Hoffman LaRoche/Sigma) was added to the wells and cell viability was measured by readout of absorbance at 450 nm and 600 nm wavelengths at saturation (Biotek Epoch plate reader), as previously described [6].

### Reverse transcription quantitative real time polymerase chain reaction (qRT-PCR) gene expression analysis

Template RNA was obtained as described in the polysome profiling section of the Supplementary Information. The RNA concentration was established by 260/280 nm wavelength absorbency measurement on NanoDrop 2000 Spectrophotometer (Thermo Fisher Scientific). Equal amounts of RNA were reverse transcribed to cDNA using High-Capacity cDNA Reverse Transcription Kit (Thermo Fisher Scientific) following manufacturer’s protocol. qRT-PCR was carried out using the QuantStudio 6 Flex RT-PCR system machine (Thermo Fisher Scientific) using commercially available FAM-labeled probes and primers (Thermo Fisher Scientific) to determine the expression of human *GATM*, *GAMT*, *CKMT1A*. *GAPDH* was used for normalization. Relative amounts of transcripts were calculated using ∆∆Ct method and graphed as fold change over transcript level in sgNT or vehicle control samples, similarly to our previous study [33].

### Statistical analysis

All statistical analyses for in vitro experiments were performed using GraphPad V.10.0 Software (Prism, La Jolla, California, USA). Statistical analyses for the mouse in vivo experiments were performed using RStudio (Posit Software, PBC, Boston, MA). Tumor volume data were analyzed using a linear mixed effects model with log_2_-transformed tumor volume as the response variable. Fixed effects included treatment group, time, and their interaction, and mouse ID was modeled as a random effect. An autoregressive (AR1) correlation structure was used to account for within-mouse correlation across time points. For survival analysis, overall survival was defined as time to euthanasia when tumor volume exceeded 2000 mm^3^. Kaplan–Meier survival curves were generated, and treatment groups were compared using the log-rank test. In vitro experiments were performed in three independent biological replicates with an average of at least two technical replicates per experiment used for mean and standard error of the mean calculations. Statistical significance was found when *p* < 0.05 unless otherwise noted.

Detailed protocols for Seahorse mitochondrial function analysis, polysome profiling, RNA sequencing, global proteomics, metabolomics, gene annotation, protein function enrichment analysis, statistical analyses and multi-omics factor analysis are provided in the Supplementary Information.

## Results

### Targeting *LARP1* results in potent antileukemic effects

**Fig. 1:**
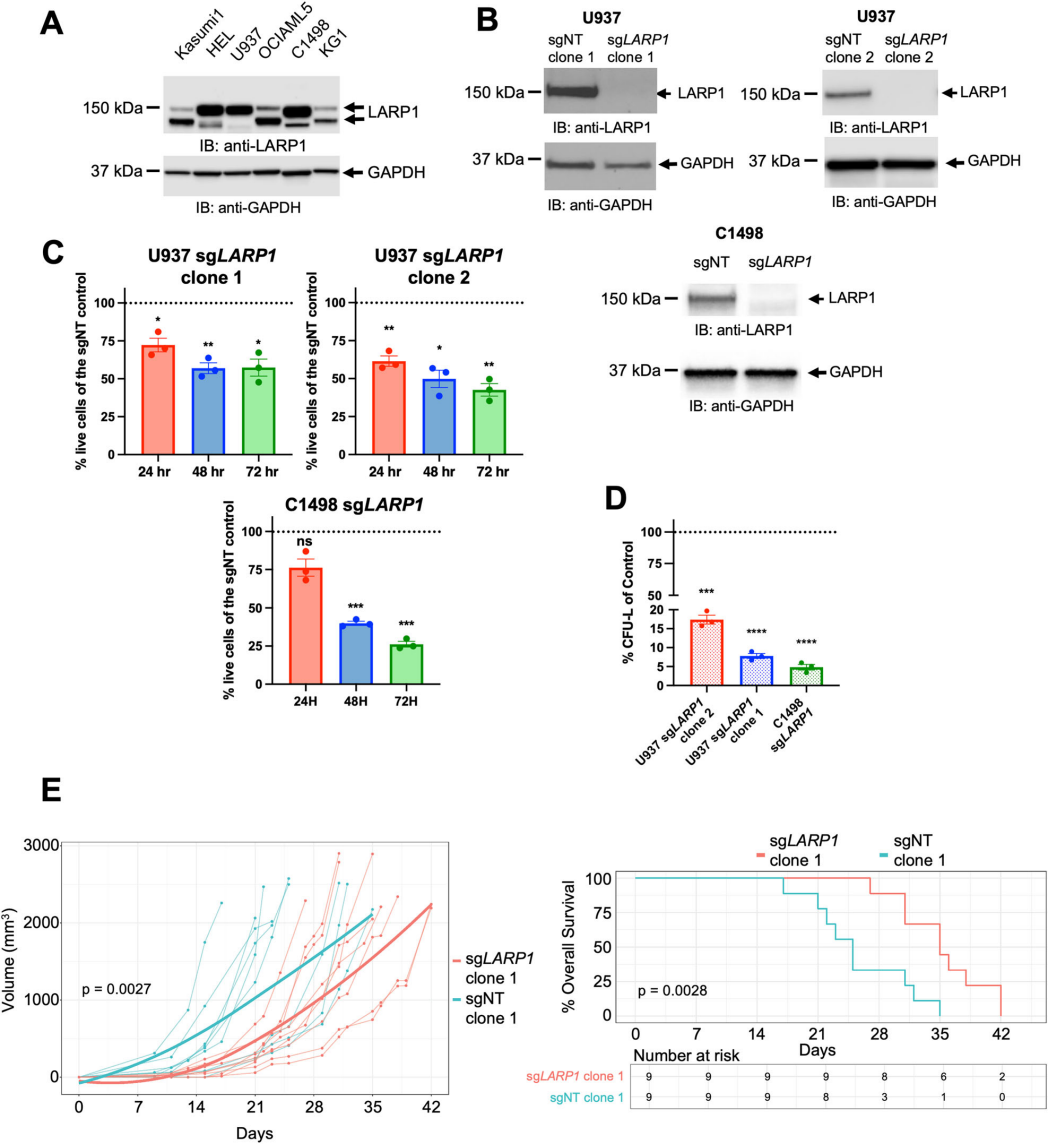
Loss of *LARP1* expression suppresses AML cell proliferation. **A** Lysates from the indicated cell lines were resolved by SDS-PAGE and immunoblotted with the indicated antibodies. **B** Single guide *LARP1* (sg*LARP1*) expressing AML cells were generated using CRISPR/Cas9 technology and validated by immunoblotting analyses. **C** Equal number of the indicated single guide non-targeting (sgNT) control and sg*LARP1* AML cells were plated and counted at the indicated time points. Trypan blue exclusion assay was used to determine cell viability. Data are normalized and expressed as a percentage live cells over sgNT control live cells within each biological replicate. Two technical replicates were used to calculate means in each biological replicate. Means ± SEM of 3 biological replicates are shown. ns – not significant, *p < 0.05, **p < 0.01, ***p < 0.001 using one-sample *t*-test for each time point compared to 100%. **D** Loss of *LARP1* expression suppresses the clonogenic ability of AML leukemic progenitors. Effects of sg*LARP1* on CFU-L leukemic progenitor colony formation from the indicated lines were assessed in clonogenic assays in methylcellulose. Data are normalized and expressed as a percentage of CFU-L units over the sgNT control within each biological replicate. Two technical replicates were used to calculate means in each biological replicate. Means ± SEM of 3 biological replicates are shown. ***p < 0.001, ****p < 0.0001 using one-sample *t*-test compared to 100%. **E** Loss of *LARP1* impairs growth of U937 xenografts in nude athymic mice. (left panel) Tumor volumes were measured at the indicated days and pooled from two independent studies (sgNT clone 1 *n* = 9, sg*LARP1* clone 1 *n* = 9); each independent study performed used 3–6 mice per group. Tumor growth was analyzed using a linear mixed effects model with log_2_-transformed tumor volume as the response variable, and fixed effects for treatment, time, and their interaction. Mouse ID was included as a random effect, and within-mouse correlation over time was modeled with an autoregressive (AR1) structure. (right panel) Kaplan–Meier survival analysis was performed using time to euthanasia (defined as tumor volume > 2000 mm^3^). *p* = 0.0028 by log-rank test.

In initial studies, we compared expression of LARP1 protein in human (Kasumi1, HEL, U937, OCIAML5, KG1) and murine (C1498) AML cell lines (Fig. 1A). HEL, U937 and C1498 cells were found to express the highest levels of LARP1 (Fig. 1A). The double band at 150/130 kDa represents the full length LARP1 (150 kDa) and an N-terminally truncated form (130 kDa) [34]. When we examined the mRNA expression of LARP1 in two independent datasets (AML TCGA [35] and BEAT AML [36] in the Bloodspot database [37] (Fig. S1A) we did not observe a significant pattern of association of *LARP1* expression consistent with any cytogenetic abnormality with the exception of −5q deleted AML due to *LARP1* being located on chromosome 5q. We next queried the LeyLab AML Proteomics Landscape (LFQ) dataset [38] and found that LARP1 protein abundance was heterogeneous across primary AML samples, with high and low expressers observed within each fusion-defined group and there was no clear fusion-restricted pattern (Fig. S1B). Altogether, these data suggest a heterogenous expression of LARP1 in AML samples without a clear association with any specific subgroup or cytogenetic abnormality.

Next, we performed gene-target deletion of *LARP1* using CRISPR/Cas9 gene editing in U937, C1498 and OCIAML5 cells. Immunoblots for selected clones of cells expressing single guide RNA targeting *LARP1* (sg*LARP1*) show complete loss of LARP1 (Fig. 1B) or partial loss of LARP1 (Fig. S2A) compared to control cells expressing single guide non-targeting RNA (sgNT). In the case of OCIAML5 (Fig. S2A), we suspect that functional p53 signaling in OCIAML5 prevented the successful selection of a clone with complete loss of *LARP1* [39, 40]. The results of subsequently performed proliferation assays indicated that the proliferation potential of sg*LARP1* U937, C1498 and OCIAML5 clones significantly decreased compared to the respective sgNT controls (Figs. 1C and S2B). Notably, loss of *LARP1* dramatically reduced the clonogenic potential of U937 and C1498 cells (Fig. 1D), with lower effects observed in OCIAML5 (Fig. S2C), possibly due to remaining low expression of LARP1 in the sg*LARP1* OCIAML5 cells (Fig. S2A).

To gain mechanistic insight into these growth defects, we examined cell cycle and apoptosis markers in U937 sg*LARP1* versus sgNT cells. LARP1 knockout consistently reduced Cyclin A2, Cyclin E1, and Aurora A protein levels (Fig. S3A). This pattern supports impaired cell cycle progression, consistent with a defect at the G1 to S transition and subsequent S-phase progression. In contrast, we did not detect increased cleavage of caspase-3 or PARP in LARP1-deficient cells (Fig. S3B), suggesting that LARP1 loss primarily limits proliferation rather than inducing overt apoptosis under steady-state culture conditions.

To assess the in vivo relevance of targeting *LARP1* on AML leukemogenesis, we implanted sg*LARP1* and sgNT U937 cells into nude mice. Tumor growth was significantly suppressed in sg*LARP1* xenografts, with reduced tumor volumes observed over time (Fig. 1E, left panel). Kaplan–Meier survival analysis indicated that mice bearing sg*LARP1* tumors had significantly prolonged survival compared to sgNT controls—median survival of 35 and 25 days, respectively (Fig. 1E, right panel). Thus, blocking LARP1 expression inhibits the growth of leukemic cells in vitro and in vivo.

### LARP1 uniquely regulates mRNA expression

**Fig. 2:**
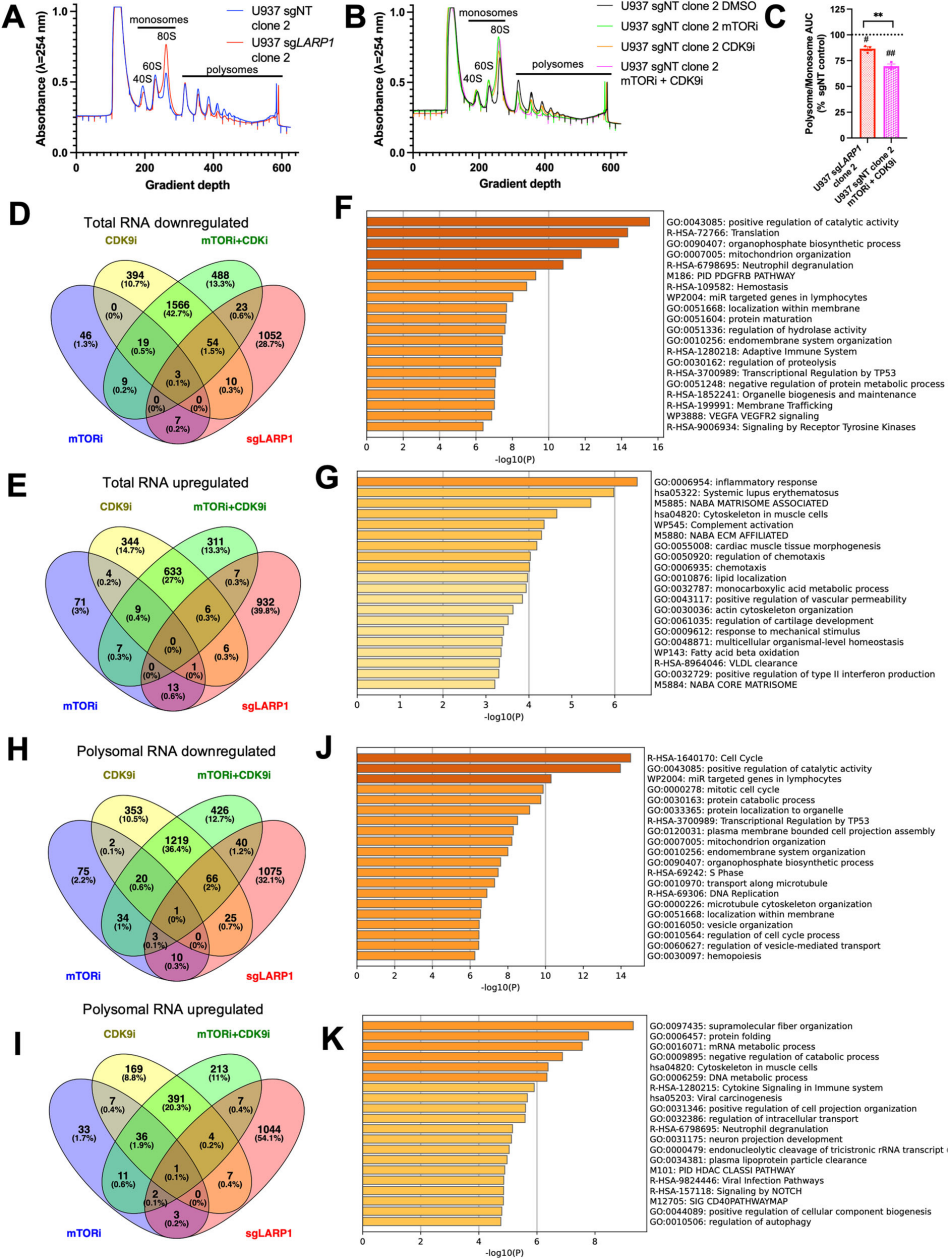
Loss of *LARP1* expression uniquely alters mRNA translation of specific genes. **A** Polysome profiles of sgNT and sg*LARP1* clone 2 U937 cells. A representative profile is shown (*n* = 3). **B** Polysome profiles of sgNT U937 clone 2 cells treated for 4 h with DMSO (vehicle), 500 nM of vistusertib (mTORi), 500 nM enitociclib (CDK9i) or their combination (mTORi + CDK9i). A representative profile is shown (*n* = 3). **C** Overall mRNA translation efficiency in U937 sg*LARP1* and U937 sgNT after mTORi and CDK9i treatment shown as ratio of the areas under the polysomal and monosomal peaks. The ratio of area under the polysomal over the monosomal peaks was calculated for each genotypic group and is represented as percent control within each biological replicate. Shown are means ± SEM of 3 biological replicates. ^#^*p* < 0.05, ^##^*p* < 0.01 using a one-sample *t*-test compared to 100%. **p < 0.01 by unpaired *t*-test. RNA-seq was performed on total (**D**–**G**) and polysomal (**H**–**K**) RNA. Venn diagrams represent the overlap in genes downregulated **D**, **H** or upregulated **E**, **I** by mTORi, CDK9i, their combination, or sg*LARP1* using a cutoff of greater than 2-fold change and *p* value < 0.05. Metascape gene ontology pathway analysis was performed for the 1052 genes significantly downregulated **F** and for the 932 genes significantly upregulated **G** at the transcriptional level uniquely in sg*LARP1* U937 cells. Metascape gene ontology pathway analysis was performed for the 1075 genes significantly downregulated **J** and the 1044 genes significantly upregulated **K** at the translational level uniquely in sg*LARP1* U937 cells.

To understand the mechanistic basis by which LARP1 regulates leukemic cell proliferation and survival, we assessed the effects of LARP1 loss on global mRNA translation. This is especially relevant, as previously published studies have suggested a paradoxical role for LARP1 in either positively or negatively regulating translation [41]. Given LARP1’s canonical role in coordinating TOP mRNA translation and ribosome biogenesis through its phosphorylation-dependent interactions with 5’ mRNA caps and ribosomes [11, 42, 43], we examined whether targeting *LARP1* would impair mRNA translation in AML cells. Polysomal profiling revealed that sg*LARP1* U937 cells exhibited a modest reduction in global translation compared to sgNT controls, with a decreased polysome-to-monosome ratio (Fig. 2A, C). We observed a greater suppression in polysome-to-monosome ratio when sgNT U937 cells were treated with both mTOR (vistusertib) and CDK9 (enitociclib) inhibitors (mTORi + CDK9i) (Fig. 2B, C). Both sg*LARP1* U937 and mTORi + CDK9i sgNT U937 cells exhibited similarly increased monosomal fractions compared to their corresponding controls. These observations support the notion that LARP1 fine-tunes rather than globally controls translation in AML cells.

To further dissect the scope of LARP1-mediated gene regulation, we performed total and polysome-associated RNA-seq in sgNT and sg*LARP1* U937 cells and compared these with sgNT U937 cells treated with mTORi, CDK9i, or their combination (Supplementary Tables 1–8 and Fig. 2D–K). Principal component analysis confirmed clear clustering by experimental group (Fig. S4), and Venn analysis revealed that knocking out *LARP1* results in unique patterns of gene expression changes, with 1052 genes downregulated and 932 genes upregulated uniquely in sg*LARP1* U937 cells compared to sgNT control, mTORi-treated, CDK9i-treated and mTORi + CDK9i-treated cells (Fig. 2D, E). Pathway enrichment analysis of the 1052 uniquely downregulated transcripts in sg*LARP1* U937 cells showed suppression of genes involved in mitochondrial gene expression, translational machinery, protein trafficking, and immune signaling (Fig. 2F and Supplementary Table 9). *LARP1*-knockout cells also showed unique upregulation of genes involved in inflammatory signaling and matrix remodeling programs (Fig. 2G and Supplementary Table 10), consistent with a potential immunomodulatory role. At the translational level, knocking out *LARP1* uniquely repressed transcripts critical for cell cycle, DNA replication, and catabolism (Fig. 2H, J and Supplementary Table 11). In contrast, translationally upregulated transcripts were associated with protein folding, mRNA metabolism, autophagy, and cytokine signaling (Fig. 2I, K and Supplementary Table 12), potentially indicative of a stress-adaptive or compensatory response. Notably, sg*LARP1* U937 cells showed nearly equal numbers of translationally up- and down- regulated genes (Fig. 2H, I), suggesting that LARP1 can act as both a suppressor and enhancer of translation depending on the context and co-factors [41, 43]. Strikingly, gene expression changes in sg*LARP1* U937 cells showed stronger overlap with CDK9 inhibition than mTOR inhibition (Figs. 2D–K and S4) [8]. These findings support LARP1 as a downstream effector that integrates CDK9 and mTOR signaling inputs to regulate leukemic gene expression products.

### Label-free quantitative proteomics reveal alterations of additional pathways upon loss of *LARP1*

**Fig. 3:**
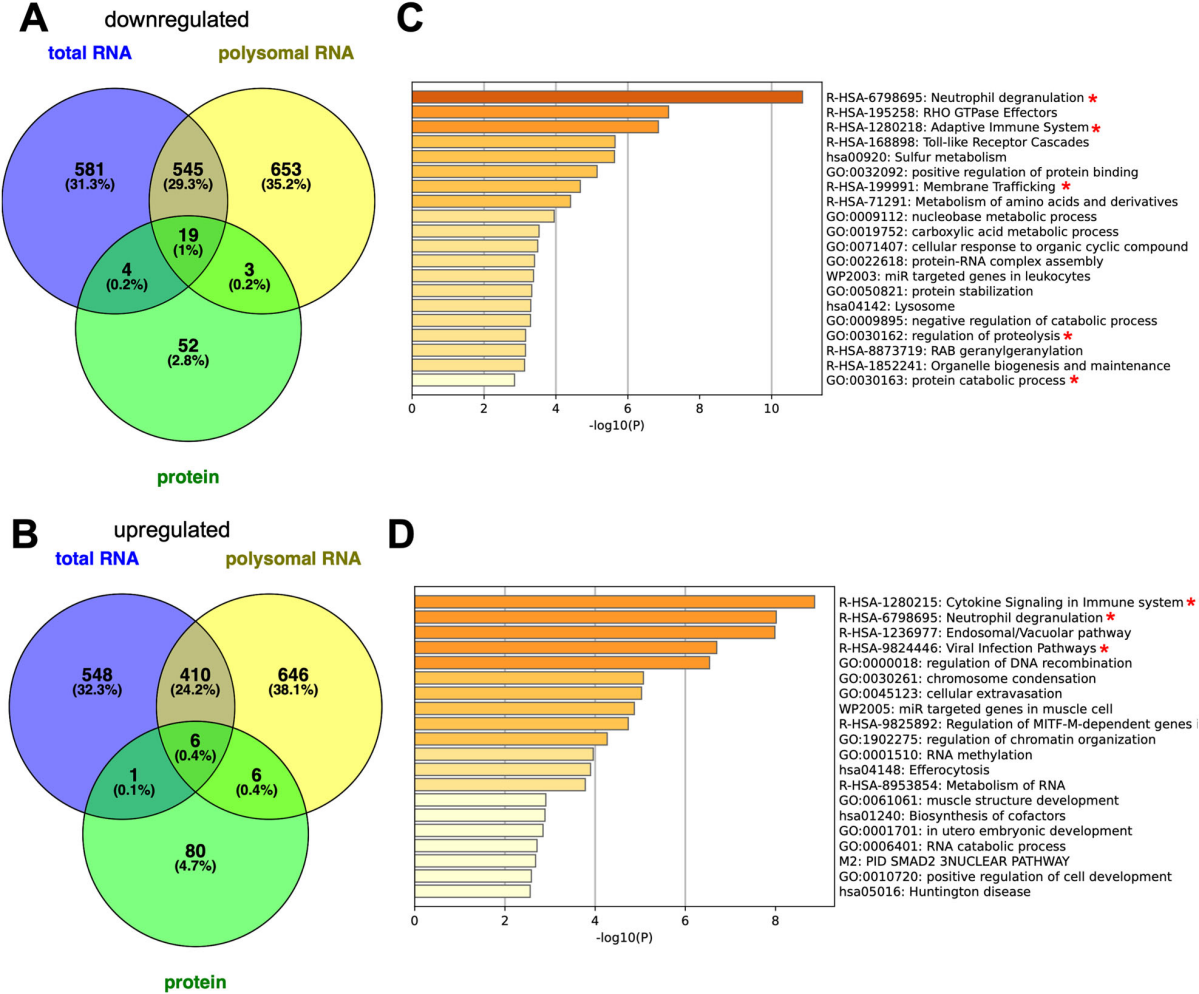
LFQ global proteomics analysis reveals pathways altered upon loss of *LARP1* expression. LFQ proteomics was performed on lysates from sg*LARP1* U937 cells (*n* = 3) and sgNT U937 cells (*n* = 2). A cutoff of 2-fold change was used to determine significantly up- or downregulated proteins because of knocking out *LARP1*. Overlap of the genes downregulated (**A**) and upregulated (**B**) in the RNA-seq analysis of total and polysomal mRNA and at the protein level in proteomics is shown. Metascape gene ontology pathway analysis was performed for the proteins found in the proteomics analysis to be significantly downregulated (**C**) and upregulated (**D**) in sg*LARP1* U937 cells compared to control sgNT cells. Asterisk indicates pathways that were also identified as altered in the RNA-seq pathway analysis.

To complement our transcriptomic and translatomic findings, we performed global label-free quantitative (LFQ) proteomics using lysates from sg*LARP1* U937 cells compared to sgNT control U937 cells (Supplementary Table 13). Integration of the proteomics data with the RNA-seq data from the same cells revealed overlapping and distinct changes in protein expression, with some protein expression altered independently of transcription or translation (Fig. 3A, B). These findings suggest that LARP1 could also influence post-translational protein stability and degradation.

Metascape pathway analysis [44] of the proteins downregulated by knocking out *LARP1* revealed that most of these are involved in immune and inflammatory signaling (e.g., neutrophil degranulation, Toll-like receptor cascades), proteostasis (protein-RNA complex assembly, proteolysis, lysosome function), amino acid and sulfur metabolism, and organelle maintenance (Fig. 3C and Supplementary Table 14). In contrast, proteins found upregulated following *LARP1* loss in U937 cells are associated with processes involved in RNA modification, chromatin organization, viral response, and cytokine signaling pathways (Fig. 3D and Supplementary Table 15). These proteomic alterations highlight a broader and more complex role for LARP1 in maintaining proteome integrity, including pathways involved in AML cell immune signaling and stress adaptation. A number of protein-level changes occurred in the absence of corresponding gene and RNA-level changes, suggesting that loss of LARP1 may affect the regulation of post-translational protein stability. This is an additional layer of regulation not previously attributed to LARP1 [43], and further studies are needed to address whether this is a direct effect of LARP1 or an indirect effect through LARP1’s regulation of mRNA stability and translation.

### Loss of *LARP1* reprograms the metabolome in AML cells

**Fig. 4:**
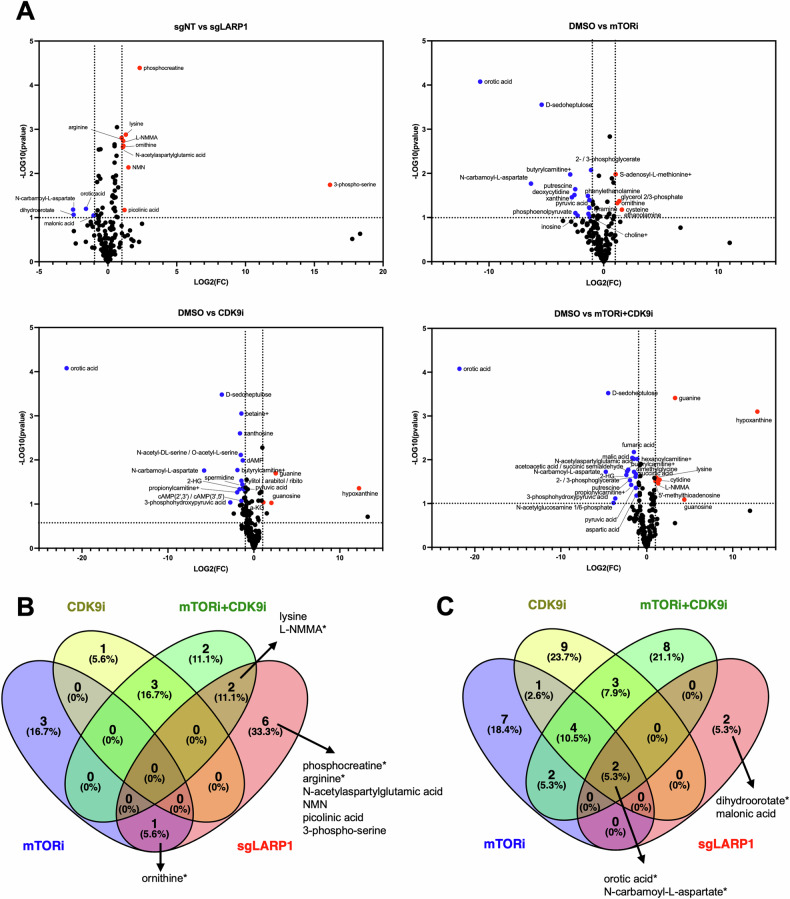
Steady state metabolomics demonstrates metabolic pathways regulated by LARP1. sgNT U937 clone 2 cells treated for 4 h with DMSO (vehicle), 500 nM of vistusertib (mTORi), 500 nM enitociclib (CDK9i) or their combination (mTORi+CDK9i). **A** Volcano plot demonstrates the significantly altered metabolites when each condition was analyzed to its matched control sample (sgNT or DMSO). A 2-fold change up or down and raw *p* value of 0.1 was used as a cutoff for significance. **B** Venn diagram shows overlap of metabolites found to be significantly upregulated compared to its control group. Asterisk indicates metabolites related to arginine metabolism. **C** Venn diagram shows overlap of metabolites found to be significantly downregulated compared to its control group. Asterisk indicates metabolites involved in pyrimidine synthesis.

Due to the observed evidence of effects on metabolic pathways in the Metascape analysis of RNA-seq and proteomics results and because of the importance of metabolic plasticity in AML pathogenesis as well as drug resistance [14], we next sought to define the effects of LARP1 on AML metabolic pathways. We performed steady-state metabolomic profiling in sg*LARP1* and sgNT U937 cells, and vehicle, mTORi, CDK9i, or their combination-treated sgNT U937 cells (Supplementary Table 16). Unsupervised visualization of the metabolomics dataset (top differentially abundant metabolite heatmaps and PCA) demonstrated consistent clustering of biological replicates and separation of conditions (Fig. S5A–D). Knocking out *LARP1* resulted in broad alterations in metabolite abundance (Fig. 4A), with upregulation of metabolites involved in the arginine and creatine metabolism pathways (e.g., arginine, ornithine, phosphocreatine, L-NMMA). Some metabolites related to arginine metabolism were elevated upon mTOR and mTOR/CDK9 inhibition (ornithine and L-NMMA). Moreover, four metabolites (N-acetylaspartylglutamic acid, NMN, picolinic acid, 3-phospho-serine) were uniquely upregulated in sg*LARP1* U937 cells (Fig. 4A, B). These results suggest specific regulation of other metabolic pathways, independently of mTOR or CDK9. We also observed shared depletion of pyrimidine biosynthesis intermediates (e.g., dihydroorotate, orotic acid) in both sg*LARP1* and mTOR and/or CDK9 inhibitor-treated U937 cells (Fig. 4A, C).

We next performed Multi-Omics Factor Analysis (MOFA) as a multimodal bioinformatic approach to further explore both shared and modality-specific sources of variation across the omics datasets (Fig. S6). The analysis used 4 types of data: input mRNA (mRNA_input), polysomal mRNA (mRNA_poly), Proteomics and Metabolomics. We used the 5000 most variable genes from the RNA-seq data, as well as the 1500 most variable peptide groups from the proteomics data and all of the metabolomic data for analysis. mRNA data were normalized according to library size, using the TMM method and logCPM values. We set the number of latent factors to four, based on the total number of samples and model diagnostics. These learned factors capture the dominant sources of variation across the omics layers and help identify different cellular states. Factors 1 and 2 are primarily driven by the RNA-seq data (Fig. S6A). Factor 2 shows a strong association with CDK9 inhibition, suggesting it represents a cellular state primarily linked to this perturbation (Fig. S6B). In contrast, Factor 1 is associated with both genotype and treatment class, prompting further investigation of the gene signatures contributing to this factor. We plotted MOFA factor 1 values vs. total mRNA input (Fig. S6C) or polysomal mRNA signal (Fig. S6D), and Factor 1 strongly correlates with global RNA abundance (both at the transcriptional and translational level). Samples treated with mTOR and/or CDK9 inhibitors largely fall along the same linear relationship as control samples, indicating that these perturbations primarily shift cells along this global RNA axis. In contrast, LARP1 knockout samples are systematically shifted toward lower total RNA and lower Factor 1 values, occupying a distinct region of this relationship. These results support the conclusion that loss of LARP1 induces a global cellular state that is not phenocopied by mTOR or CDK9 inhibition alone. This interpretation is further supported by heatmaps of gene expression for genes most strongly associated with Factor 1 in the polysomal mRNA data, as plotted in both mRNA input (Fig. S6E) and mRNA polysomal data (Fig. S6F).

**Fig. 5:**
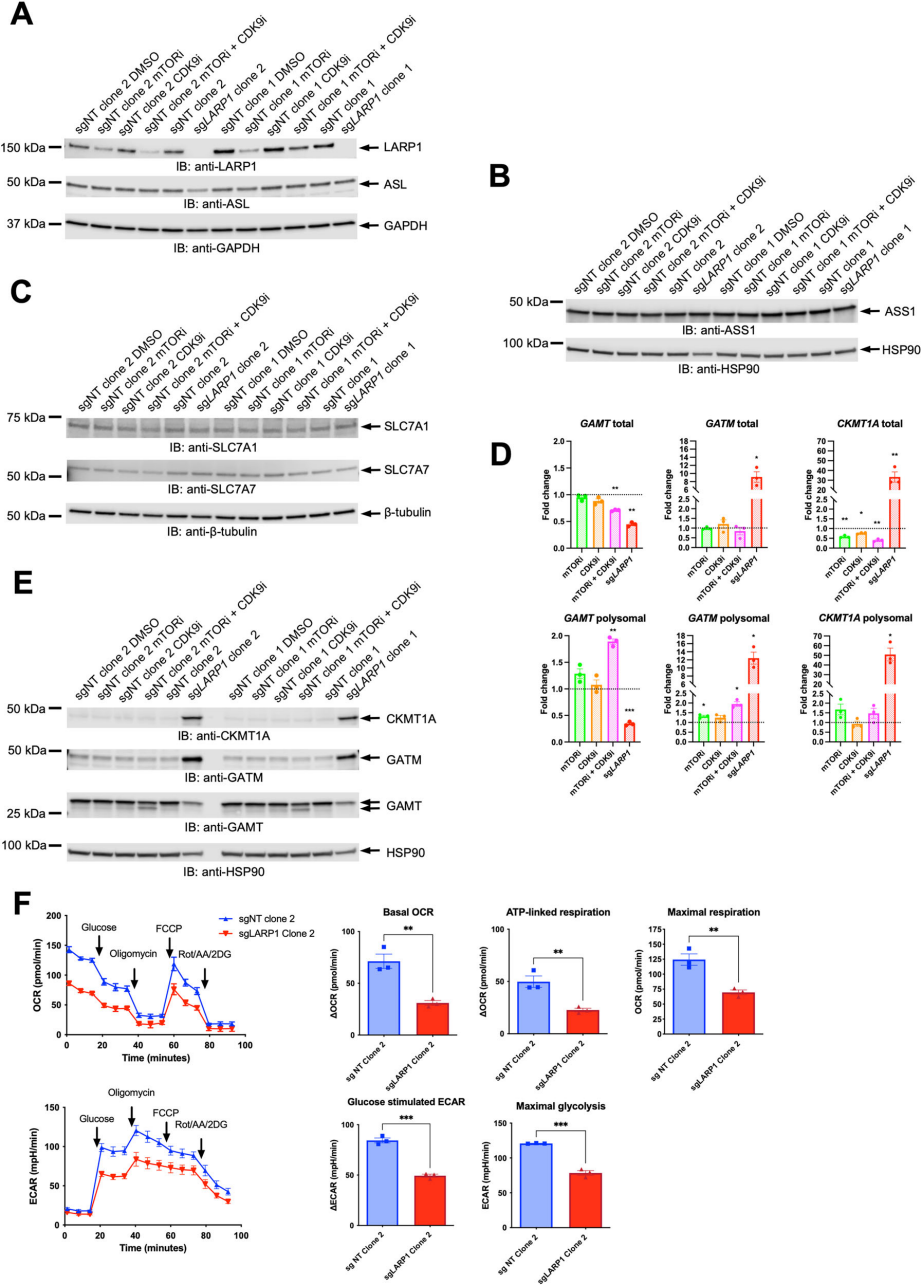
Effects of LARP1 on arginine-creatine metabolic pathways and mitochondrial/glycolytic function in AML cells. **A**–**C** Lysates of sgNT U937 clones 1 and 2 cells treated for 4 h with DMSO (vehicle), 500 nM of vistusertib (mTORi), 500 nM enitociclib (CDK9i) or their combination (mTORi + CDK9i), sg*LARP1* U937 clone 1 and 2 cells and sgNT U937 clone 1 and 2 cells were resolved by SDS-PAGE and immunoblotted with the indicated antibodies. **D** qRT-PCR was performed on total and polysomal RNA for each respective condition—sgNT U937 clone 2 cells treated for 4 h with DMSO (vehicle), 500 nM of vistusertib (mTORi), 500 nM enitociclib (CDK9i) or their combination (mTORi + CDK9i), sg*LARP1* U937 clone 2 cells and sgNT U937 clone 2 cells. Data are expressed as fold change over each sets respective control (DMSO or sgNT) within each biological replicate. *GAPDH* was used for normalization. Means ± SEM of three biological replicates are shown. *p < 0.05, **p < 0.01, ***p < 0.001 using a one-sample *t*-test compared to 1.0-fold change. **E** Cell lysates were resolved by SDS-PAGE and immunoblotted with the indicated antibodies. sgNT U937 clone 2 cells were treated for 4 h with DMSO (vehicle), 500 nM of vistusertib (mTORi), 500 nM enitociclib (CDK9i) or their combination (mTORi+CDK9i). **F** Mitochondrial respiration and glycolytic activity in sgNT and sg*LARP1* U937 clone 2 cells were assessed using a Seahorse XF Cell Mito Stress Test assay. OCR and ECAR were measured at baseline and after sequential injections of glucose, oligomycin, FCCP, and a mixture of rotenone/antimycin A with 2-deoxy-D-glucose (Rot/AA/2DG). Shown are representative OCR and ECAR traces from one biological experiment (mean ± SEM of five technical wells per condition). Bar graphs summarize basal respiration, ATP-linked respiration and maximal respiration, as well as glucose-stimulated ECAR and maximal glycolysis, calculated from three independent biological experiments and presented as mean ± SEM. **p < 0.01, ***p < 0.001 by unpaired *t*-test between the indicated groups.

In further studies, we sought to validate the key findings from our omics data and uncover the mechanisms whereby LARP1 regulates key cellular metabolic pathways. We investigated if there were any differences in the levels of ASS1, ASL, SLC7A1 and SLC7A7 proteins, involved in the arginine metabolic pathway, comparing sg*LARP1* to sgNT U937 cells, and mTOR and/or CDK9 inhibitor-treated cells. We did not observe any striking changes at the protein level for these enzymes and transporters between experimental conditions (Fig. 5A–C), suggesting that the increased arginine levels in *LARP1* KO U937 cells were not due to altered expression of these genes, but possibly due to changes in the function of the corresponding proteins.

We next examined whether LARP1 loss affects the expression of the creatine metabolism pathway components. We observed an increase in *GATM* and *CKMT1A* expression in the RNA-seq and a decrease in *GAMT* in RNA-seq and LFQ Proteomics analysis (Figs. 2 and 3). We validated the findings by qRT-PCR and immunoblotting analyses and demonstrate that GATM and CKMT1A expression are greatly increased and GAMT expression is decreased at both the protein and RNA level (Fig. 5D, E). These alterations were either minor or not observed in the mTOR and/or CDK9-treated cells. Together with our metabolomics data, these results suggest that LARP1 exerts transcript-specific regulation of key and specific proteins involved in the phosphocreatine pathway, leading to a shift in the creatine cycle balance.

To directly assess mitochondrial function, we performed a Seahorse XF Cell Mito Stress Test in U937 clone 2 sgNT and sg*LARP1* cells. LARP1 knockout cells showed a significant reduction in basal oxygen consumption rate (OCR), ATP-linked respiration and maximal respiratory capacity compared with sgNT controls (Fig. 5F), indicating impaired oxidative phosphorylation. Glycolytic parameters from extracellular acidification rate (ECAR) were also significantly reduced in sg*LARP1* versus sgNT cells. LARP1 loss impairs both mitochondrial oxidative phosphorylation and glycolysis, suggesting that LARP1 may contribute to overall metabolic function in AML cells.

**Fig. 6:**
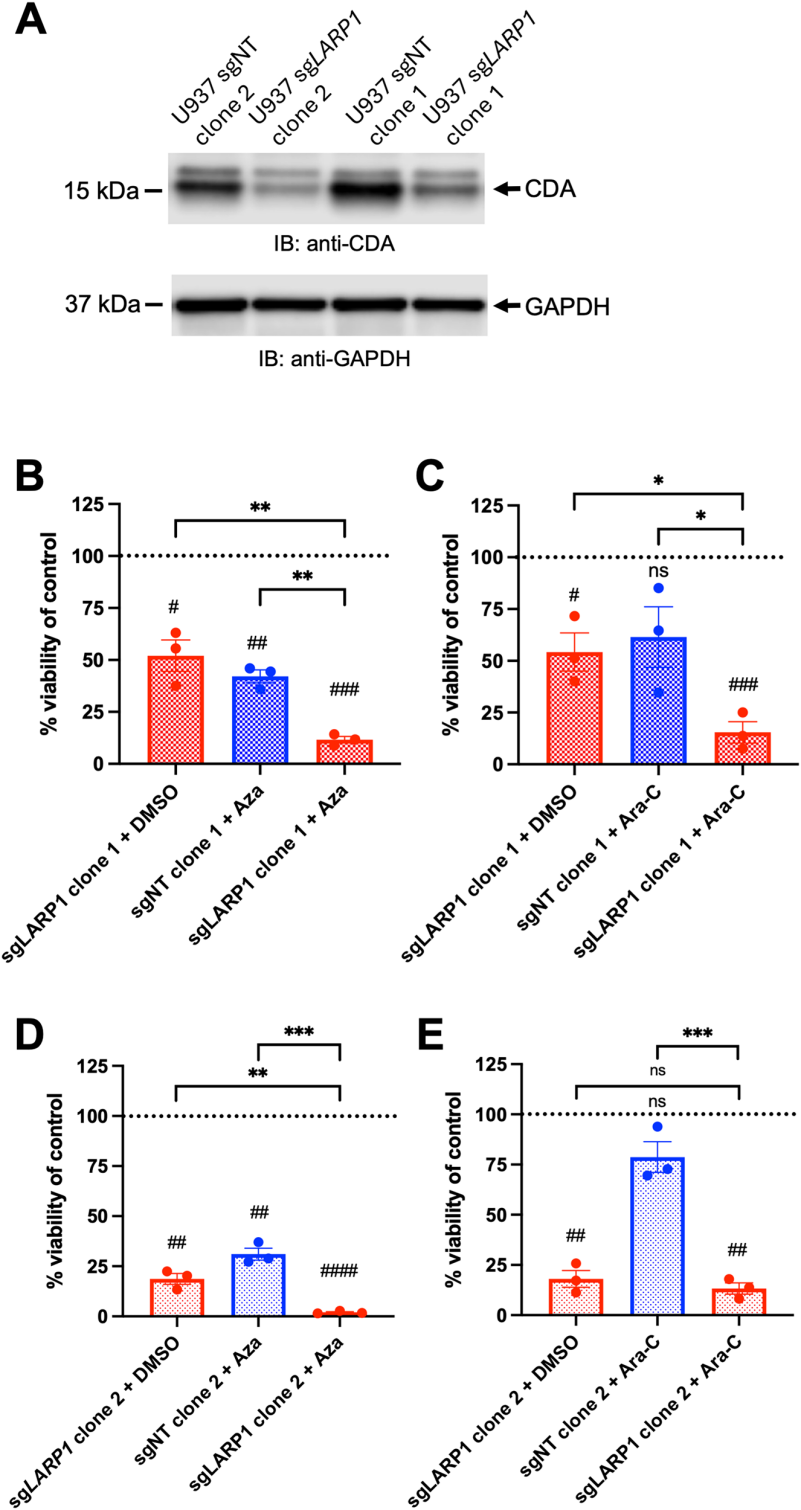
Loss of *LARP1* expression enhances the antileukemic effects of 5-azacytidine and cytarabine. **A** Cell lysates from the indicated U937 clones were resolved by SDS-PAGE and immunoblotted with the indicated antibodies. Equal number of sgNT and sg*LARP1* U937 clone 1 (**B**, **C**) and clone 2 (**D**, **E**) cells were treated for 4 days with 5-azacytidine (Aza, 1 μM) (**B**, **D**) or cytarabine (Ara-C, 3 ng/ml) (**C**, **E**) followed by WST assays to assess cell viability. Data are normalized and expressed as a percentage of viability over DMSO-treated sgNT cells (sgNT + DMSO) within each biological replicate. Means ± SEM of 3 biological replicates are shown. A one-sample *t*-test compared to 100% for vehicle treated (DMSO) samples ns—not significant, ^#^*p* < 0.05, ^##^*p* < 0.01, ^###^*p* < 0.001, ^####^*p* < 0.0001. A one-way ANOVA with Holm-Sidak multiple comparison adjustment was used to compare drug-treated groups * adjusted *p* < 0.05, ** adjusted *p* < 0.01.

Finally, we prioritized cytidine deaminase (CDA) as a follow-up hit because it emerged as a reproducibly LARP1-regulated gene across our multi-omics layers and has direct pharmacologic relevance to standard AML nucleoside analog therapy. CDA is a pyrimidine metabolism enzyme and a known resistance factor for cytarabine and 5-azacytidine, as it metabolizes nucleoside analogs to inactive metabolites [45–47]. We found that the expression of this enzyme was significantly downregulated both in the RNA-seq and LFQ proteomics analysis upon knocking out *LARP1* (Figs. 2 and 3) and we further validated these findings by immunoblotting (Fig. 6A). Importantly, there was a statistically significant enhancement of azacitidine and cytarabine efficacy in *LARP1*-knockout cells (Fig. 6B–E), supporting a role for LARP1 as a potential driver of metabolic drug resistance mechanisms and as a promising novel target to enhance sensitivity to these drugs in AML.

## Discussion

Our study identifies LARP1 as a critical and previously underappreciated regulator of gene expression and metabolic homeostasis in AML. Through an integrative multi-omics approach, we show that LARP1 plays essential roles in leukemic cell proliferation, metabolic rewiring, and resistance to chemotherapy. These functions are potentially mediated through LARP1’s capacity to selectively control mRNA translation, stabilize transcripts, and regulate protein abundance [11–13]—activities that are distinct from and only partially overlapping with those of mTOR and CDK9 signaling. Importantly, the dominant pathway-level outputs are consistent across omics layers. LARP1 loss most reproducibly impacts mitochondrial function, translation/ribosome programs, cell cycle/DNA replication, stress/inflammatory signaling, and nucleotide and amino acid metabolism. These signatures intersect known mTOR/CDK9-linked biology, supporting LARP1 as a downstream effector, while also highlighting selective remodeling of the leukemic cell state that is not fully recapitulated by mTOR/CDK9 inhibition. This could be either due to compensatory kinases that can activate LARP1 when mTOR or CDK9 are inhibited or LARP1 can regulate unique signaling pathways irrespective of its phosphorylation status.

Functionally, targeting *LARP1* suppressed proliferation in human and murine AML cell lines, reduced clonogenic capacity, and impaired tumor growth and increased survival in an AML xenograft model. These findings establish LARP1 as an essential element for AML maintenance and progression. High *LARP1* expression has similarly been associated with progression in solid tumors, including ovarian, colorectal, and liver cancer [21, 24, 48].

LARP1 loss significantly enhanced the cytotoxicity of standard AML therapies, such as 5-azacytidine and cytarabine. Mechanistically, this was associated with reduced expression of cytidine deaminase at both the transcript and protein level, potentially limiting drug inactivation [45–47]. We highlight CDA based on its consistent LARP1-dependent regulation across matched experimental models and its established pharmacologic relevance. These findings indicate that LARP1 may play a key role in mediating metabolic drug resistance and indicate that LARP1 targeting can potentially enhance the clinical efficacy of existing anti-leukemia agents.

At the molecular level, polysome profiling revealed that *LARP1* knockout moderately reduced global mRNA translation, though to a lesser extent than pharmacological inhibition of mTOR or CDK9. Thus, unlike mTOR or CDK9, which broadly impact the translational machinery, LARP1 exhibits more selective translational effects. RNA-sequencing analysis demonstrated distinct transcriptomic and translatomic changes upon *LARP1* loss, with only partial overlap with mTOR or CDK9 inhibition. Notably, knocking out *LARP1* caused nearly equal numbers of translationally up- and downregulated transcripts, further supporting a model in which LARP1 can act both as a positive and negative regulator of mRNA translation. The direction of LARP1-mediated translational control may depend on the cellular context, post-translational modifications, or specific mRNA sequence elements. These findings highlight the nuanced regulatory role of LARP1 and suggest that its contribution to mRNA translation is likely shaped by both upstream signaling and transcript-specific interactions, rather than a simple binary function downstream of mTOR or CDK9. Consistent with the reduced proliferation, LARP1 knockout decreased Cyclin A2, Cyclin E1 and Aurora A protein levels without increasing basal caspase-3 or PARP cleavage, suggesting that impaired cell cycle progression, rather than strong induction of apoptosis, is the dominant consequence of LARP1 loss in this setting.

Steady-state metabolomics demonstrated that LARP1 plays a central role in AML cellular metabolic pathways. Notably, *LARP1*-knockout cells also displayed decreased levels of pyrimidine biosynthesis intermediates, a phenotype shared with mTOR/CDK9 inhibitor-treated cells, linking LARP1 to nucleotide metabolism. Given that mTORC1 controls pyrimidine synthesis via S6K1 [49] and LARP1 has been shown to modulate this output [13] our findings suggest that LARP1 functions as an important component of a metabolic regulatory hub, integrating upstream signaling inputs with control of nucleotide and amino acid metabolism. Additionally, targeting *LARP1* led to the accumulation of arginine pathway metabolites (e.g., arginine, ornithine, phosphocreatine). The arginine metabolic pathway is often dysregulated in AML through loss of ASS1 and/or OTC expression and dependence on extracellular arginine [17]. *LARP1* knockout cells exhibited upregulation of arginine and arginine pathway metabolites. While RNA-seq and proteomics demonstrated changes in amino acid metabolism, the mechanism for arginine accumulation remains unclear, as protein-level changes in metabolic enzymes or arginine transporters were not detected. Pegylated-Arginase BCT-100 has activity in AML cells due to depletion of arginine, leading to cell death and less immunosuppression and has been recently tested in clinical trials [16, 17, 19]. ADI-PEG 20 is another drug used to target exogenous arginine dependence that also has activity in AML and has advanced to clinical trials [16, 18, 50]. Further studies will be required to determine if LARP1 targeting can increase sensitivity to either of these potential AML therapies and/or if it can synergize with other arginine pathway modulators, such as drugs that target amino acid transporters.

Interestingly, we also observed transcript-specific effects on the phosphocreatine synthesis pathway. GAMT was consistently downregulated at both transcript and translational levels, while GATM and CKMT1A were upregulated, suggesting that LARP1 fine-tunes energy buffering pathways rather than uniformly suppressing or promoting their activity. These findings underscore the context-dependence of LARP1’s translational control, as previously proposed [41, 43]. As phosphocreatine can act as an energy store to convert ADP to ATP in times of high energy needs [51, 52], the increase in phosphocreatine could be a survival mechanism upon *LARP1* loss. Consistent with this notion, our Seahorse analysis shows that LARP1-deficient AML cells have significantly impaired oxidative phosphorylation and glycolytic capacity, indicating a global deficit in energy production. In this setting, the increased phosphocreatine levels observed in our metabolomic analysis likely reflect compensatory engagement of the creatine kinase/phosphocreatine system as an energy buffer when mitochondrial capacity is compromised, rather than enhanced bioenergetic fitness. The creatine kinase pathway has previously been shown to be a target to alter cellular metabolism in EVI1-positive AML cells through inhibiting CKMT1A [51]. Further studies will be required to determine if targeting this metabolic pathway will lead to a specific vulnerability upon *LARP1* loss. Additionally, future studies with syngeneic or humanized mouse models will be required to determine the role LARP1 plays in regulating the interplay between AML cells and other cells in the bone marrow microenvironment through alteration of translation and metabolic pathways and evaluate the combinatorial effects of targeting LARP1 with standard AML treatment modalities.

Taken together, our data position LARP1 as a key integrator of gene expression, translation and metabolism in AML. By linking mRNA stability, selective translation, and protein regulation, LARP1 supports oncogenic programs essential for leukemic cell survival. These findings support further development of LARP1 targeting strategies in AML. Its influence extends beyond canonical mTOR and CDK9 outputs, and its loss exposes vulnerabilities in metabolic pathways that could be leveraged for therapeutic targeting.

## Supplementary information

Supplementary Information

Table S1

Table S2

Table S3

Table S4

Table S5

Table S6

Table S7

Table S8

Table S9

Table S10

Table S11

Table S12

Table S13

Table S14

Table S15

Table S16

Original data file - western blots

Original data file - qPCR CT values

## Data Availability

RNA-seq raw data files have been uploaded to the NIH GEO web portal under the accession number GSE304358. LFQ Proteomics raw data have been uploaded to the PRIDE database under accession number PXD064460. All other data are available upon request. For original data, please contact LCP (l-platanias@northwestern.edu) or EMB (e-beauchamp@northwestern.edu).

## Code availability

The code used for data analysis was uploaded to the GitHub web portal. Code used for RNA-seq data analysis is accessible at: https://github.com/brianlee9267/RNAseq-LARP1-Analysis, and the code used for MOFA analysis is accessible at: https://github.com/brianlee9267/MOFA-LARP1-Analysis.

## References

[1] Dohner H, Weisdorf DJ, Bloomfield CD. Acute myeloid leukemia. N Engl J Med. 2015;373:1136–52. 26376137 10.1056/NEJMra1406184

[2] Dohner H, Wei AH, Appelbaum FR, Craddock C, DiNardo CD, Dombret H, et al. Diagnosis and management of AML in adults: 2022 recommendations from an international expert panel on behalf of the ELN. Blood. 2022;140:1345–77. 35797463 10.1182/blood.2022016867

[3] Kantarjian H, Short NJ, DiNardo C, Stein EM, Daver N, Perl AE, et al. Harnessing the benefits of available targeted therapies in acute myeloid leukaemia. Lancet Haematol. 2021;8:e922–33. 34687602 10.1016/S2352-3026(21)00270-2 PMC8996707

[4] Numan Y, Abaza Y, Altman JK, Platanias LC. Advances in the pharmacological management of acute myeloid leukemia in adults. Expert Opin Pharmacother. 2022;23:1535–43. 35938317 10.1080/14656566.2022.2111212 PMC9648129

[5] Dinner S, Platanias LC. Targeting the mTOR pathway in leukemia. J Cell Biochem. 2016;117:1745–52. 27018341 10.1002/jcb.25559

[6] Colamonici M, Blyth G, Saleiro D, Szilard A, Bliss-Moreau M, Giles FJ, et al. Dual targeting of acute myeloid leukemia progenitors by catalytic mTOR inhibition and blockade of the p110alpha subunit of PI3 kinase. Oncotarget. 2015;6:8062–70. 25823922 10.18632/oncotarget.3509 PMC4480735

[7] Carneiro BA, Kaplan JB, Altman JK, Giles FJ, Platanias LC. Targeting mTOR signaling pathways and related negative feedback loops for the treatment of acute myeloid leukemia. Cancer Biol Ther. 2015;16:648–56. 25801978 10.1080/15384047.2015.1026510 PMC4622839

[8] Beauchamp EM, Abedin SM, Radecki SG, Fischietti M, Arslan AD, Blyth GT, et al. Identification and targeting of novel CDK9 complexes in acute myeloid leukemia. Blood. 2019;133:1171–85. 30587525 10.1182/blood-2018-08-870089 PMC6418475

[9] Philippe L, Vasseur JJ, Debart F, Thoreen CC. La-related protein 1 (LARP1) repression of TOP mRNA translation is mediated through its cap-binding domain and controlled by an adjacent regulatory region. Nucleic Acids Res. 2020;48:7604–5. 32558918 10.1093/nar/gkaa535 PMC7367174

[10] Lahr RM, Fonseca BD, Ciotti GE, Al-Ashtal HA, Jia JJ, Niklaus MR, et al. La-related protein 1 (LARP1) binds the mRNA cap, blocking eIF4F assembly on TOP mRNAs. Elife. 2017;6:e24146. 28379136 10.7554/eLife.24146 PMC5419741

[11] Philippe L, van den Elzen AMG, Watson MJ, Thoreen CC. Global analysis of LARP1 translation targets reveals tunable and dynamic features of 5’ TOP motifs. Proc Natl Acad Sci USA. 2020;117:5319–28. 32094190 10.1073/pnas.1912864117 PMC7071917

[12] Ogami K, Oishi Y, Sakamoto K, Okumura M, Yamagishi R, Inoue T, et al. mTOR- and LARP1-dependent regulation of TOP mRNA poly(A) tail and ribosome loading. Cell Rep. 2022;41:111548. 36288708 10.1016/j.celrep.2022.111548

[13] Hong S, Freeberg MA, Han T, Kamath A, Yao Y, Fukuda T, et al. LARP1 functions as a molecular switch for mTORC1-mediated translation of an essential class of mRNAs. Elife. 2017;6:e25237. 28650797 10.7554/eLife.25237 PMC5484620

[14] Mishra SK, Millman SE, Zhang L. Metabolism in acute myeloid leukemia: mechanistic insights and therapeutic targets. Blood. 2023;141:1119–35. 36548959 10.1182/blood.2022018092 PMC10375271

[15] Jones CL, Stevens BM, D’Alessandro A, Reisz JA, Culp-Hill R, Nemkov T, et al. Inhibition of amino acid metabolism selectively targets human leukemia stem cells. Cancer Cell. 2019;35:333–5. 30753831 10.1016/j.ccell.2019.01.013 PMC6389327

[16] Chen C, Zhang J. Enhancing leukemia treatment: the role of combined therapies based on amino acid starvation. Cancers. 2024;16:1171. 38539506 10.3390/cancers16061171 PMC10969718

[17] Mussai F, Egan S, Higginbotham-Jones J, Perry T, Beggs A, Odintsova E, et al. Arginine dependence of acute myeloid leukemia blast proliferation: a novel therapeutic target. Blood. 2015;125:2386–96. 25710880 10.1182/blood-2014-09-600643 PMC4416943

[18] Miraki-Moud F, Ghazaly E, Ariza-McNaughton L, Hodby KA, Clear A, Anjos-Afonso F, et al. Arginine deprivation using pegylated arginine deiminase has activity against primary acute myeloid leukemia cells in vivo. Blood. 2015;125:4060–8. 25896651 10.1182/blood-2014-10-608133

[19] Mussai F, De Santo C, Abu-Dayyeh I, Booth S, Quek L, McEwen-Smith RM, et al. Acute myeloid leukemia creates an arginase-dependent immunosuppressive microenvironment. Blood. 2013;122:749–58. 23733335 10.1182/blood-2013-01-480129 PMC3731930

[20] Wetzel TJ, Erfan SC, Figueroa LD, Wheeler LM, Ananieva EA. Crosstalk between arginine, glutamine, and the branched chain amino acid metabolism in the tumor microenvironment. Front Oncol. 2023;13:1186539. 37274280 10.3389/fonc.2023.1186539 PMC10235471

[21] Ye L, Lin ST, Mi YS, Liu Y, Ma Y, Sun HM, et al. Overexpression of LARP1 predicts poor prognosis of colorectal cancer and is expected to be a potential therapeutic target. Tumour Biol. 2016;37:14585–94. 27614686 10.1007/s13277-016-5332-3 PMC5126195

[22] Xie C, Huang L, Xie S, Xie D, Zhang G, Wang P, et al. LARP1 predict the prognosis for early-stage and AFP-normal hepatocellular carcinoma. J Transl Med. 2013;11:272. 24159927 10.1186/1479-5876-11-272 PMC3814951

[23] Mura M, Hopkins TG, Michael T, Abd-Latip N, Weir J, Aboagye E, et al. LARP1 post-transcriptionally regulates mTOR and contributes to cancer progression. Oncogene. 2015;34:5025–36. 25531318 10.1038/onc.2014.428 PMC4430325

[24] Ma J, Dong D, Qi H, Li J, Yu H, Hu X, et al. LARP1, an RNA-binding protein, participates in ovarian cancer cell survival by regulating mitochondrial oxidative phosphorylation in response to the influence of the PI3K/mTOR pathway. Biochim Biophys Acta Mol Basis Dis. 2024;1870:167453. 39111634 10.1016/j.bbadis.2024.167453

[25] Zhao R, Yang L, Liu C, Jiang R, Huang Q, Wang Q, et al. A novel N7-Methylguanine-related gene signature for predicting prognosis in acute myeloid leukemia: bioinformatic analysis and experimental verification. Hematology. 2024;29:2433905. 39611741 10.1080/16078454.2024.2433905

[26] Sriskanthadevan S, Jeyaraju DV, Chung TE, Prabha S, Xu W, Skrtic M, et al. AML cells have low spare reserve capacity in their respiratory chain that renders them susceptible to oxidative metabolic stress. Blood. 2015;125:2120–30. 25631767 10.1182/blood-2014-08-594408 PMC4375109

[27] Wu S, Fahmy N, Alachkar H. The mitochondrial transcription machinery genes are upregulated in acute myeloid leukemia and associated with poor clinical outcome. Metabol Open. 2019;2:100009. 32812906 10.1016/j.metop.2019.100009 PMC7424792

[28] Firmanty P, Chomczyk M, Dash S, Konopleva M, Baran N. Feasibility and safety of targeting mitochondria function and metabolism in acute myeloid leukemia. Curr Pharmacol Rep. 2024;10:388–404. 40756330 10.1007/s40495-024-00378-8 PMC12314886

[29] Hsu PP, Kang SA, Rameseder J, Zhang Y, Ottina KA, Lim D, et al. The mTOR-regulated phosphoproteome reveals a mechanism of mTORC1-mediated inhibition of growth factor signaling. Science. 2011;332:1317–22. 21659604 10.1126/science.1199498 PMC3177140

[30] Jia JJ, Lahr RM, Solgaard MT, Moraes BJ, Pointet R, Yang AD, et al. mTORC1 promotes TOP mRNA translation through site-specific phosphorylation of LARP1. Nucleic Acids Res. 2021;49:3461–89. 33398329 10.1093/nar/gkaa1239 PMC8034618

[31] Parmar S, Smith J, Sassano A, Uddin S, Katsoulidis E, Majchrzak B, et al. Differential regulation of the p70 S6 kinase pathway by interferon alpha (IFNalpha) and imatinib mesylate (STI571) in chronic myelogenous leukemia cells. Blood. 2005;106:2436–43. 15790787 10.1182/blood-2004-10-4003 PMC1895266

[32] Small SH, Perez RE, Beauchamp EM, Baran AH, Willis SD, Fischietti M, et al. Targeting SLFN11-regulated pathways restores chemotherapy sensitivity in AML. Blood Neoplasia. 2024;1:100037. 40552140 10.1016/j.bneo.2024.100037 PMC12182837

[33] Saleiro D, Kosciuczuk EM, Fischietti M, Perez RE, Yang GS, Eckerdt F, et al. Targeting CHAF1B enhances IFN activity against myeloproliferative neoplasm cells. Cancer Res Commun. 2023;3:943–51. 37377894 10.1158/2767-9764.CRC-23-0010 PMC10231401

[34] Schwenzer H, Abdel Mouti M, Neubert P, Morris J, Stockton J, Bonham S, et al. LARP1 isoform expression in human cancer cell lines. RNA Biol. 2021;18:237–47. 32286153 10.1080/15476286.2020.1744320 PMC7928056

[35] Cancer Genome Atlas Research N, Ley TJ, Miller C, Ding L, Raphael BJ, Mungall AJ, et al. Genomic and epigenomic landscapes of adult de novo acute myeloid leukemia. N Engl J Med. 2013;368:2059–74. 23634996 10.1056/NEJMoa1301689 PMC3767041

[36] Tyner JW, Tognon CE, Bottomly D, Wilmot B, Kurtz SE, Savage SL, et al. Functional genomic landscape of acute myeloid leukaemia. Nature. 2018;562:526–31. 30333627 10.1038/s41586-018-0623-z PMC6280667

[37] Gislason MH, Demircan GS, Prachar M, Furtwangler B, Schwaller J, Schoof EM, et al. BloodSpot 3.0: a database of gene and protein expression data in normal and malignant haematopoiesis. Nucleic Acids Res. 2024;52:D1138–42. 37933860 10.1093/nar/gkad993 PMC10768446

[38] Kramer MH, Zhang Q, Sprung R, Day RB, Erdmann-Gilmore P, Li Y, et al. Proteomic and phosphoproteomic landscapes of acute myeloid leukemia. Blood. 2022;140:1533–48. 35895896 10.1182/blood.2022016033 PMC9523374

[39] Gentilella A, Moron-Duran FD, Fuentes P, Zweig-Rocha G, Riano-Canalias F, Pelletier J, et al. Autogenous control of 5’TOP mRNA stability by 40S ribosomes. Mol Cell. 2017;67:55–70. e4. 28673543 10.1016/j.molcel.2017.06.005 PMC5553558

[40] Prokocimer M, Molchadsky A, Rotter V. Dysfunctional diversity of p53 proteins in adult acute myeloid leukemia: projections on diagnostic workup and therapy. Blood. 2017;130:699–712. 28607134 10.1182/blood-2017-02-763086 PMC5659817

[41] Berman AJ, Thoreen CC, Dedeic Z, Chettle J, Roux PP, Blagden SP. Controversies around the function of LARP1. RNA Biol. 2021;18:207–17. 32233986 10.1080/15476286.2020.1733787 PMC7928164

[42] Saba JA, Huang Z, Schole KL, Ye X, Bhatt SD, Li Y, et al. LARP1 binds ribosomes and TOP mRNAs in repressed complexes. EMBO J. 2024;43:6555–72. 39533057 10.1038/s44318-024-00294-z PMC11649897

[43] Deragon JM, Bousquet-Antonelli C. The role of LARP1 in translation and beyond. Wiley Interdiscip Rev RNA. 2015;6:399–417. 25892282 10.1002/wrna.1282

[44] Zhou Y, Zhou B, Pache L, Chang M, Khodabakhshi AH, Tanaseichuk O, et al. Metascape provides a biologist-oriented resource for the analysis of systems-level datasets. Nat Commun. 2019;10:1523. 30944313 10.1038/s41467-019-09234-6 PMC6447622

[45] Gu X, Tohme R, Tomlinson B, Sakre N, Hasipek M, Durkin L, et al. Decitabine- and 5-azacytidine resistance emerges from adaptive responses of the pyrimidine metabolism network. Leukemia. 2021;35:1023–36. 32770088 10.1038/s41375-020-1003-x PMC7867667

[46] Donnette M, Hamimed M, Ciccolini J, Sicard G, Correard F, Farnault L, et al. Cytidine deaminase status as a marker of response to azacytidine treatment in MDS and AML patients. Br J Haematol. 2023;203:625–36. 37691342 10.1111/bjh.19096

[47] Fajardo-Orduna GR, Ledesma-Martinez E, Aguiniga-Sanchez I, Mora-Garcia ML, Weiss-Steider B, Santiago-Osorio E. Inhibitors of chemoresistance pathways in combination with Ara-C to overcome multidrug resistance in AML. A mini review. Int J Mol Sci. 2021;22:4955. 34066940 10.3390/ijms22094955 PMC8124548

[48] Zhu WL, Zeng H, Huang DP, Ouyang WJ, Wei CS, Tong GD. The potential impact of EIF4E3 and LARP1 on tumor immunity in hepatocellular carcinoma. Biomed Environ Sci. 2023;36:469–75. 37253674 10.3967/bes2023.057

[49] Ben-Sahra I, Howell JJ, Asara JM, Manning BD. Stimulation of de novo pyrimidine synthesis by growth signaling through mTOR and S6K1. Science. 2013;339:1323–8. 23429703 10.1126/science.1228792 PMC3753690

[50] Tsai HJ, Jiang SS, Hung WC, Borthakur G, Lin SF, Pemmaraju N, et al. A phase II study of arginine deiminase (ADI-PEG20) in relapsed/refractory or poor-risk acute myeloid leukemia patients. Sci Rep. 2017;7:11253. 28900115 10.1038/s41598-017-10542-4 PMC5595917

[51] Fenouille N, Bassil CF, Ben-Sahra I, Benajiba L, Alexe G, Ramos A, et al. The creatine kinase pathway is a metabolic vulnerability in EVI1-positive acute myeloid leukemia. Nat Med. 2017;23:301–13. 28191887 10.1038/nm.4283 PMC5540325

[52] Kazak L, Cohen P. Creatine metabolism: energy homeostasis, immunity and cancer biology. Nat Rev Endocrinol. 2020;16:421–36. 32493980 10.1038/s41574-020-0365-5

