# Supplementary material for: Multi-omics analysis reveals LARP1 as a key integrator of translation and metabolism in AML
Source: Oncogenesis. 2026 May 16;15(1):37. doi: 10.1038/s41389-026-00623-3 (PMC13346483; doi:10.1038/s41389-026-00623-3)
Supplement: Supplementary file 1 — Supplementary Information [file 41389_2026_623_MOESM1_ESM.pdf]

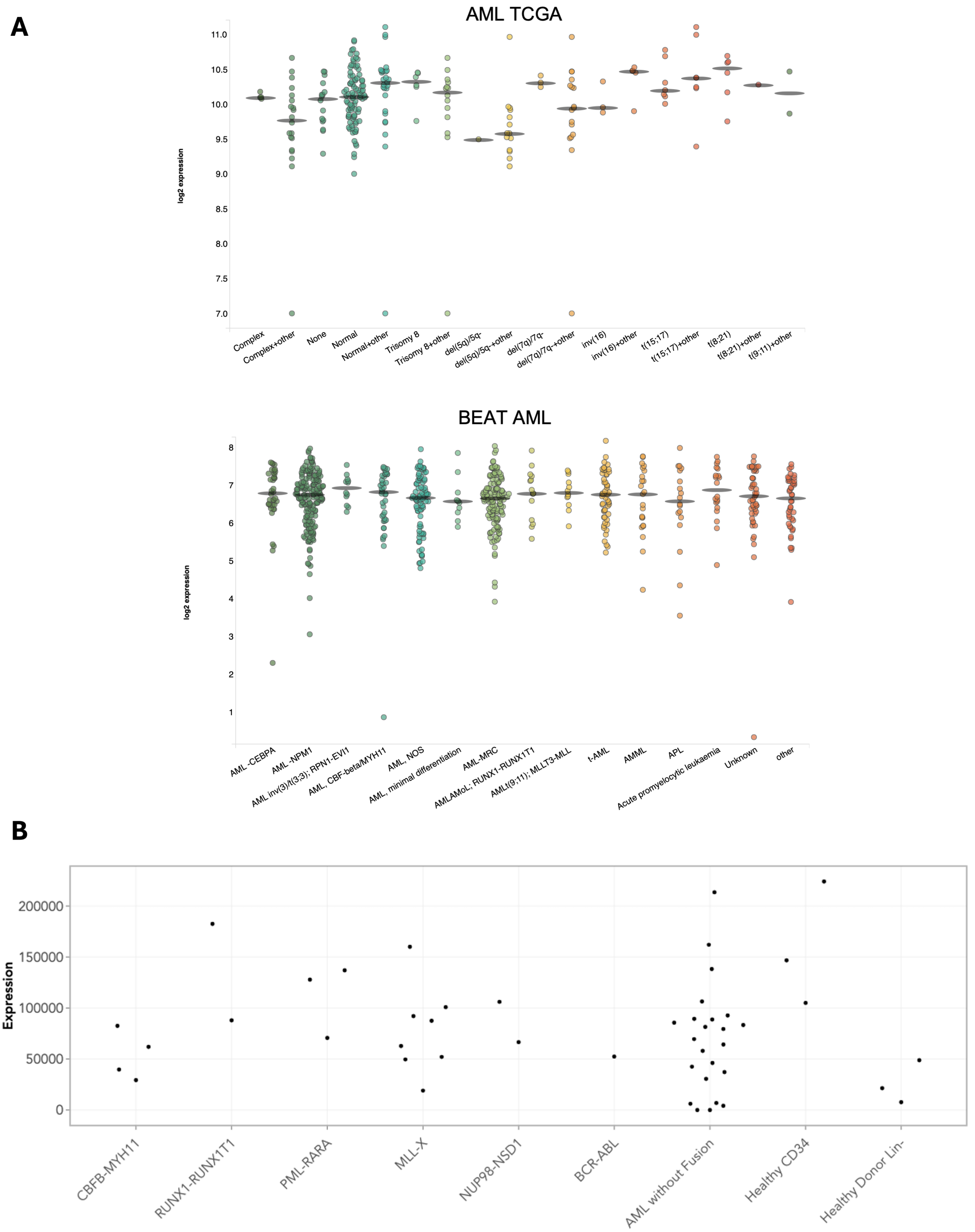

Fig. S1. LARP1 expression across AML molecular subgroups from publically available TCGA and proteomic databases. **A** Relative LARP1 mRNA expression across different AML cytogenetic subgroups in the TCGA AML and Beat AML datasets, obtained from BloodSpot. **B** LARP1 protein abundance levels of AML patient samples stratified by key fusion events and compated to normal hematopoietic controls. Data were obtained from the LeyLab AML Proteomics Landscape database.

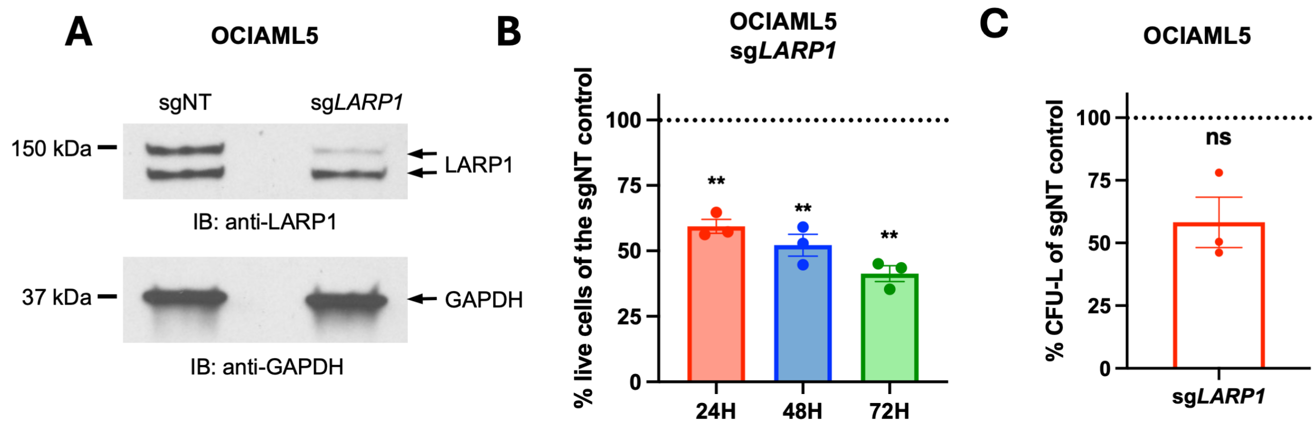

Fig. S2. Loss of *LARP1* expression suppresses AML cell growth. **A** Single guide *LARP1* (sg*LARP1*) expressing OCIAML5 cells were generated using CRISPR/CAS9 technology and validated by immunoblotting analysis compared to sgNT control OCIAML5 cells. **B** Equal number of sgNT control and sg*LARP1* OCIAML5 cells were plated and counted at the indicated time points. Trypan blue exclusion assay was used to determine cell viability. Data are expressed as percentage live cells over sgNT control live cells. Means ± SEM of 3 biological replicates are shown. ** $p < 0.01$ using one sample t test for each time point compared to 100%. **C** Loss of *LARP1* expression suppresses the clonogenic ability of OCIAML5 leukemic progenitors. Effects of sg*LARP1* on CFU-L leukemic progenitor colony was assessed in clonogenic assays in methylcellulose. Data are expressed as percentage of CFU-L units over the sgNT control within each biological replicate. Means ± SEM of 3 biological replicates are shown. ns – nonsignificant using one sample t test compared to control at 100%.

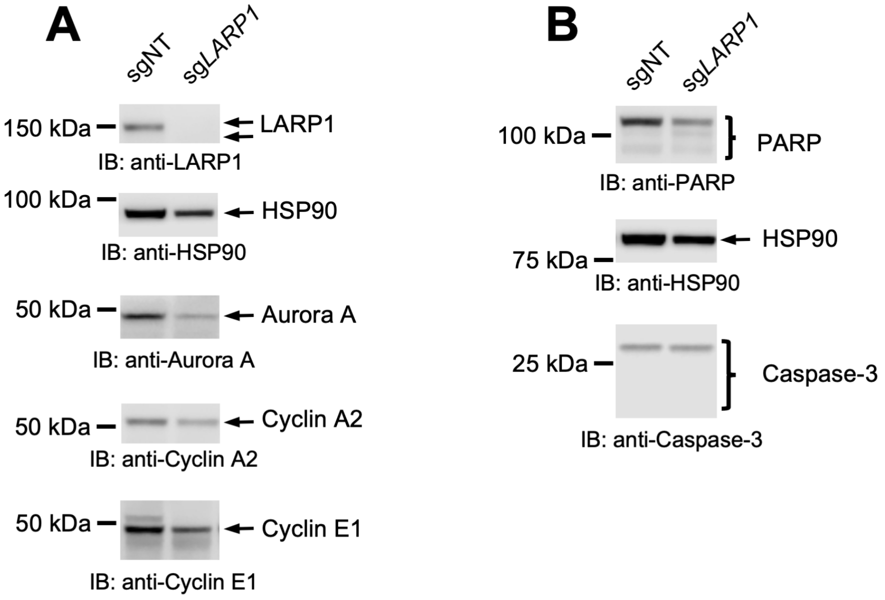

Fig. S3. Effects of *LARP1* expression loss on **A** cell cycle and **B** apoptosis. Cell lysates from U937 clone 2 cells expressing single guide RNA targeting *LARP1* (sg*LARP1*) or non targeting control (sgNT) were resolved by SDS-PAGE and immunoblotted with the indicated antibodies
.

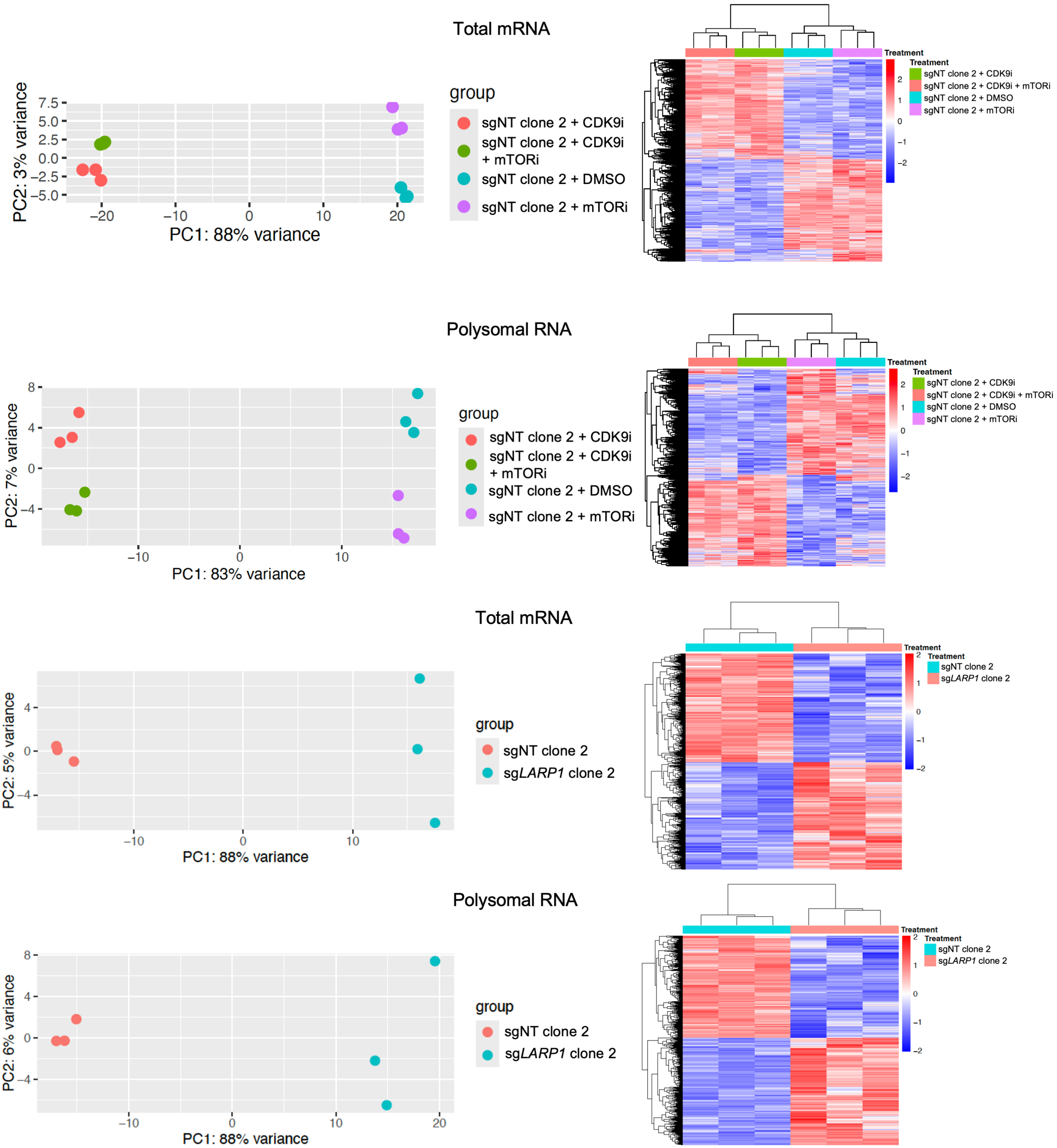

Fig. S4. PCA and Heatmaps for the RNA-seq analysis demonstrating clustering of the samples for each experimental group. Polysome profiling was performed for sg*LARP1* U937 clone 2, sgNT U937 clone 2 cells (n=3), and sgNT U937 clone 2 cells treated with vehicle control (DMSO), 500 nM of vistusertib (mTORi), 500 nM enitociclib (CDK9i) or their combination (mTORi+CDK9i) for 4 hours (n=3). Total and polysome-associated RNA was subjected to RNA-seq.

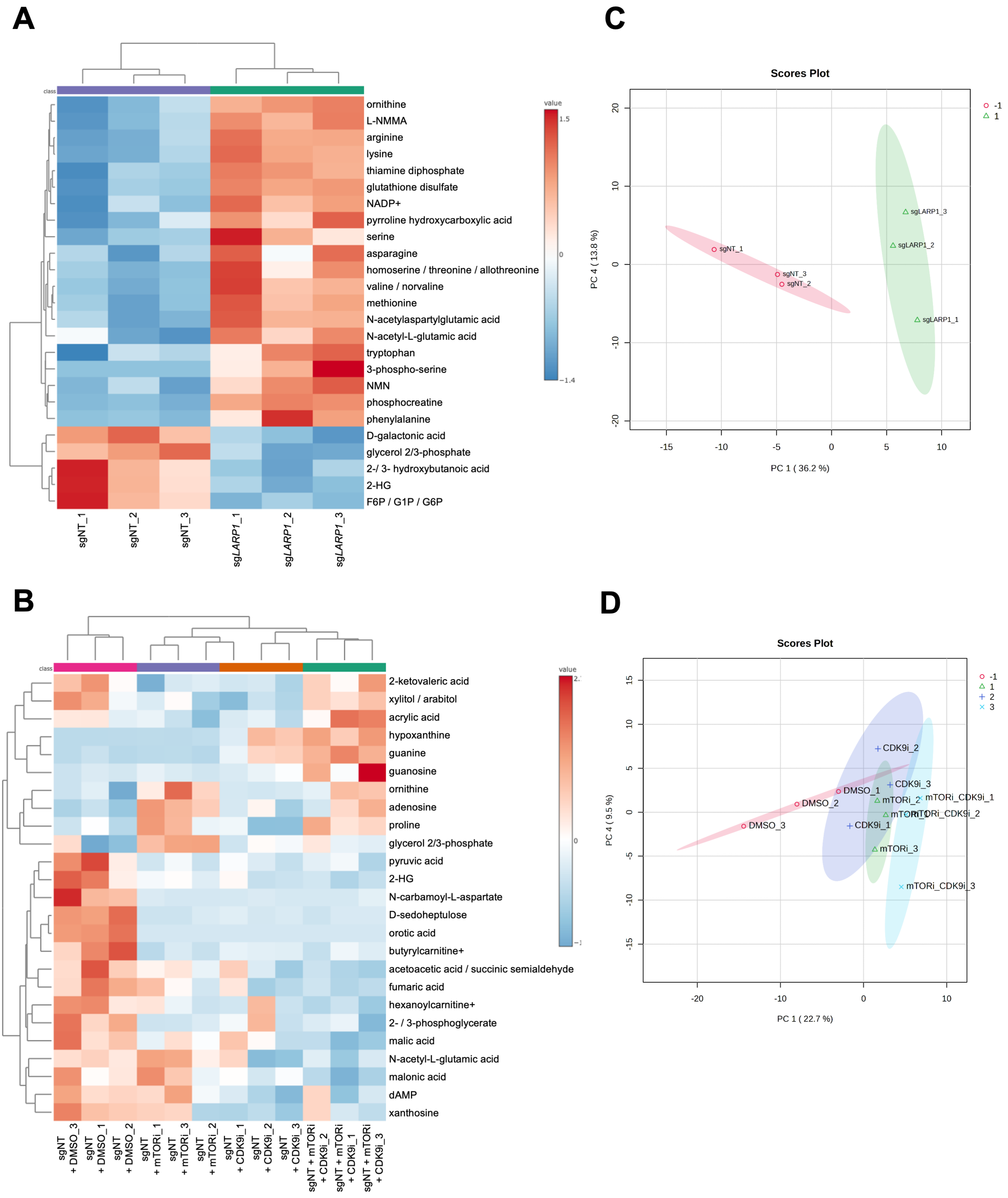

Fig. S5. Heatmap shows top 25 differentially regulated metabolites (up or down) using a **A** t-test or **B** One-way ANOVA for each triplicate in each experimental condition **C** and **D** PCA analysis demonstrating clustering of the different samples for each experimental group. Steady state LC-MS based metabolomics was performed for sg*LARP1* U937 clone 2, sgNT U937 clone 2 cells (n=3), and sgNT U937 clone 2 cells treated with vehicle control (DMSO), 500 nM of vistusertib (mTORi), 500 nM enitociclib (CDK9i) or their combination (mTORi+CDK9i) for 4 hours (n=3).

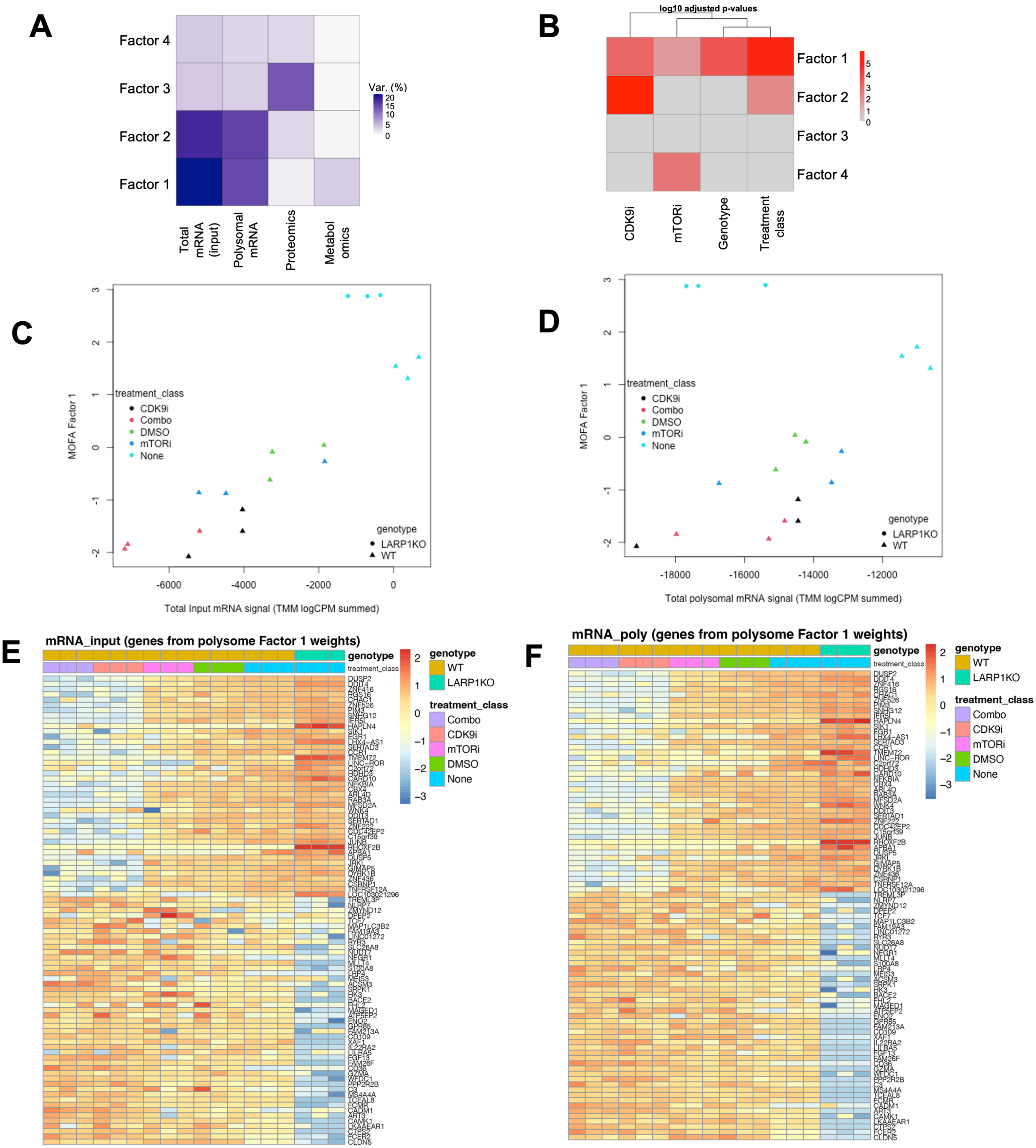

Fig. S6. Integrated multi-omics factor analysis (MOFA) reveals that loss of LARP1 induces a distinct global cellular state relative to mTOR and/or CDK9 inhibition. MOFA was performed using the MOFA2 R package, by integrating total (input) and polysome-associated RNA-seq data, metabolomics data, generated from U937 clone 2 sgNT and sgLARP1 cells, together with sgNT cells treated for 4 h with vehicle (DMSO), 500 nM vistusertib (mTORi), 500 nM enitociclib (CDK9i), or their combination (mTORi+CDK9i), and proteomics data generated from U937 clone 2 sgNT and sgLARP1 cells. **A** Fraction of variance explained by MOFA factors 1-4 within each omics layer (total mRNA, polysomal mRNA, proteomics, metabolomics). **B** Association of each factor with experimental covariates (CDK9i, mTORi, genotype, treatment class) shown as log10 adjusted p-values (higher/red indicates stronger association). **C-D** Relationship between MOFA Factor 1 values and global total input **C** or polysomal **D** mRNA signal (summed TMM-normalized logCPM) for each sample; points are colored by treatment class and shaped by genotype illustrating that LARP1 KO samples are systematically shifted toward lower RNA abundance and lower Factor 1 values, deviating from the pattern seen for inhibitor-treated and control samples. **E-F**. Heatmaps of genes most significantly correlated with Factor 1 based on polysomal RNA-seq weights, visualized across total input **E** and polysomal **F** mRNA datasets, revealing coordinated transcriptional and translational changes associated with this latent factor.

Key Resources Table

REAGENT OR RESOURCE	SOURCE	IDENTIFIER
**Antibodies**		
LARP1 (D8J4F) Rabbit mAb	Cell Signaling Technology	Cat#: 70180<br>RRID:AB_2799778
ASS1 antibody	Cell Signaling Technology	Cat#: 70720<br>RRID:AB_2799790
ASL antibody	Proteintech	Cat#: 16645-1-AP<br>RRID:AB_2878293
SLC7A1 (CAT-1) antibody	Proteintech	Cat#: 14195-1-AP<br>RRID:AB_2190723
SLC7A7 antibody	Proteintech	Cat#: 84743-1-RR<br>RRID:AB_3672128
CKMT1A antibody	Proteintech	Cat#: 15346-1-AP<br>RRID:AB_2081073
GATM antibody	Proteintech	Cat#: 12801-1-AP<br>RRID:AB_2109060
GAMT antibody	Proteintech	Cat#: 10880-1-AP<br>RRID:AB_2109304
CDA Polyclonal antibody	Proteintech	Cat#: 28579-1-AP<br>RRID:AB_2881174
AURKA Monoclonal antibody	Proteintech	Cat#: 66757-1-Ig<br>RRID:AB_2882103
Cyclin A2 (BF683) Mouse Monoclonal Antibody	Cell Signaling Technology	Cat#: 4656S<br>RRID:AB_2071958
Cyclin E1 (D7T3U) Rabbit mAb	Cell Signaling Technology	Cat#: 20808T<br>RRID:AB_2783554
PARP Antibody	Cell Signaling Technology	Cat#: 9542S<br>RRID:AB_2160739
Caspase-3 Antibody	Cell Signaling Technology	Cat#: 9662S<br>RRID:AB_331439
Anti-Glyceraldehyde-3-Phosphate Dehydrogenase Antibody, clone 6C5	MilliporeSigma	Cat#: MAB374<br>RRID:AB_2107445
HSP 90alpha/beta (F-8)	Santa Cruz Biotechnology	Cat#: sc-13119<br>RRID:AB_675659
Goat Anti-Mouse IgG (H + L)-HRP Conjugate	BioRad	Cat#: 1706516<br>RRID:AB_2921252
Anti-rabbit IgG, HRP-linked Antibody	Cell Signaling Technology	Cat#: 7074<br>RRID:AB_2099233
Precision Protein StrepTactin-HRP Conjugate	BioRad	Cat#:1610381
**Experimental models: cell lines**		
U937	ATCC	Cat#: CRL-1593.2<br>RRID:CVCL_0007
OCI-AML-5	DSMZ	Cat#: ACC 247<br>RRID:CVCL_1620

C1498	ATCC	Cat#: C1498-TIB-49<br>RRID:CVCL_3494
Kasumi-1	ATCC	Cat#: CRL-2724<br>RRID:CVCL_0589
HEL	ATCC	Cat#: TIB-180<br>RRID:CVCL_2481
KG-1	ATCC	Cat#: CCL-246<br>RRID:CVCL_0374
**Experimental models: mice**		
CrTac:NCr-Foxn1^{nu}	Taconic Biosciences	Cat#: NCRNU-F<br>RRID:IMSR_TAC:NCRNU
**Taqman probes**		
GATM	Thermo Fisher Scientific	Cat#: 4331182<br>Assay ID: Hs00933793_m1
GAMT	Thermo Fisher Scientific	Cat#: 4331182<br>Assay ID: Hs00355745_g1
CKMT1A	Thermo Fisher Scientific	Cat#: 4331182<br>Assay ID: Hs00179727_m1
GAPDH	Thermo Fisher Scientific	Cat#: 4331182<br>Assay ID: Hs03929097_g1
**Chemicals, peptides, recombinant proteins**		
RPMI	Gibco	Cat#: 11875119
MEM alpha	Gibco	Cat#: 12571063
DMEM	Gibco	Cat#: 11965118
FBS	Sigma	Cat#: F2442-500ML
OptiMEM	Gibco	Cat#: 31985070
MethoCult Classic	STEMCELL Technologies	Cat#: H4534
MethoCult GF	STEMCELL Technologies	Cat#: M3434
Sodium pyruvate 100 mM	Gibco	Cat#: 11965118
Sodium bicarbonate 7.5%	Gibco	Cat#: 25080094
Trypan Blue Dye 0.4%	BioRad	Cat#: 1450013
Puromycin 10 mg/mL	Gibco	Cat#: 1113803
MycoAlert Mycoplasma detection kit	Lonza	Cat#: LT07-218
Lenti-X Packaging Single Shots (VSV-G)	Takara	Cat#: 631275
Spinoculation kit (TransDux MAX, SBI #LV860-A1)	SBI	Cat#: LV860-A1
Lenti-X concentrator kit	Takara	Cat#: 631232
Quantitative lentiviral titer test Lenti-X GoStix Plus	Takara	Cat#: 631280
Bicinchonic acid assay	Pierce	Cat#: 23224<br>Cat#: 23223
Precision Plus Protein Dual Color Standards	BioRad	Cat#: 1610374
Precision Plus Protein WesternC Standards BioRad	BioRad	Cat#:1610385

Lane Marker Reducing Sample Buffer 5x	Thermo Scientific	Cat#: 39000
BioRad Criterion TGX Precast 4-15% Gels	BioRad	12-well Cat#: 5671083 18-well Cat#: 5671084
10x Tris/Glycine/SDS running buffer	National Diagnostics	Cat#: EC-870
Trans-Blot Turbo RTA Midi 0.45 µm LF PVDF Transfer Kit	BioRad	Cat#: 1704275
Nonfat dry milk	BioRad	Cat#: 1706404
GE Healthcare Amersham™ ECL™	GE Healthcare	Cat#: 45-000-875
Biorad Clarity Max ECL Western Blotting Substrate	BioRad	Cat#: 1705062
WesternSure® PREMIUM Chemiluminescent Substrate	LiCORbio	Cat#: 926-95000
HyBlot CL Autoradiography Film	Thomas Scientific	Cat#: 1141J52
GM-CSF	PeproTech	Cat#: 300-03
WST-1	Hoffman LaRoche / Sigma	Cat#: 5015944001
5-azacitidine	TargetMol	Cat#: T1272
Cytarabine	TargetMol	Cat#: T1339
Vistusertib (AZD-2014)	Chemietek	Cat#: CT-A2014
Enitociclib (BAY -1251152)	MedChemExpress	Cat#: HY-103019B
Cycloheximide	Thermo Scientific Chemicals	Cat#: J66901.03
Protector RNase Inhibitor	Sigma / Roche	Cat#: 3335402001
Protease Inhibitor Cocktail III	Sigma	Cat#: 539134-10ML
Protease Inhibitor Cocktail V	Sigma	Cat#: 539137-10VL
Sucrose	Sigma	Cat#: RDD023-1KG
Heparin sodium salt from porcine intestinal mucosa	Sigma	Cat#: H3393-100KU
Qiagen AllPrep RNA/Protein Kit	Qiagen	Cat#: 80404
High Capacity cDNA Reverse Transcription Kit	Thermo Fisher Scientific	Cat#: 4368813
**Edit-R All-in-one sgRNA system for CRISPR/Cas9 KO**		
Targeting human *LARP1* (lentivirus particles)	Horizon (previously Dharmacon)	Cat#: VSGH11936-247722211
Targeting mouse *LARP1* (lentivirus particles)	Horizon (previously Dharmacon)	Cat#: VSGM11942-247831291
Non-Targeting Control plasmid (human and mouse) – glycerol stock	Horizon (previously Dharmacon)	Cat#: GSGC11953
LB Agar Plates with Carbenicillin-100mg/mL	Teknova	Cat#: L1010
One Shot™ OmniMAX™ 2 T1R Chemically Competent E. coli	Thermo Fisher Scientific (Invitrogen)	Cat#: C854003

Endotoxin-free plasmid midiprep DNA purification-NucleoBond Xtra Midi EF	Takara (Macherey-Nagel)	Cat#: 740420.50
NucleoSpin® Plasmid (No Lid)	Takara (Macherey-Nagel)	Cat#: 740499.250
Seahorse XF mitochondrial function assay components		
Seahorse XF Cell Mito Stress Test Kit	Agilent	Cat#: 103015-100
Seahorse FluxPaks	Agilent	Cat#: 103792-100
Seahorse XF Media & Calibrant	Agilent	Cat#: 103335-100
Corning™ Cell-Tak Cell and Tissue Adhesive	Thermo Fisher Scientific (Corning)	Cat#: CB-40240
**Software and Algorithms**		
GraphPad Prism	GraphPad software Inc	https://www.graphpad.com/scientific-software/prism/ RRID:SCR_002798
Metascape 3.5		http://metascape.org/ RRID:SCR_016620
Excel	Microsoft	N/A
ImageJ	Adobe	https://imagej.net/ij/ RRID:SCR_003070
LI-COR Empiria Studio	LI-CORbio	https://www.licorbio.com/empiria-studio RRID:SCR_022512
Biorad Image Lab	Biorad	https://www.bio-rad.com/en-us/product/image-lab-software?ID=KRE6P5E8Z
QuantStudio Real-Time PCR Software	Thermo Fisher Scientific	https://www.fishersci.com/shop/products/6-flx96wfast-instlptp-1-system/4485699 RRID:SCR_020239

CRISPR / CRISPR associated protein 9 (Cas9) gene editing (additional details)

To generate cell lines expressing Cas9 and sgRNA targeting *LARP1*, $2 \cdot 10^6$ cells were transduced with Dharmacon Edit-R All-in-one Lentiviral sgRNA particles (Horizon). Control cells (Cas9 + sgNT) were generated using in-house produced lentivirus from the Edit-R Non-Targeting Control plasmid (Horizon, glycerol stock), packaged using Lenti-X Singel Shots (VSV-G, Takara) following manufacturer's instructions. Virus-containing media were harvested from transfected 293T cells, concentrated via PEG precipitation (Lenti-X Concentrator, Takara), and resuspended in OptiMEM (Gibco). Lentiviral titers were measured using the Lenti-X GoStix Plus kit (Takara). AML cell lines

were transduced at MOI 0.3 using TransDux MAX spinoculation (SBI), followed by puromycin selection the next day. Clones were established by serial dilution. To support low-density growth, cells were cultured in a 1:1 mix of fresh and conditioned media. Knockout efficiency was assessed by immunoblotting.

Immunoblotting (additional details)

Cells were harvested by centrifugation and lysed in Triton-X 100 lysis buffer (40 mM HEPES, 120 mM NaCl, 1 mM EDTA, 10 mM NaPP, 50 mM NaF, 0.5% Triton-X 100, 10 mM β-glycerophosphate) supplemented with 1 mM phenylmethylsulfonylfluoride, protease inhibitor cocktail set V (EMD Millipore), and phosphatase inhibitor cocktail set I (EMD Millipore). Protein concentrations were measured using the BCA assay (Pierce). Samples were prepared in Lane Marker Reducing Sample Buffer (Thermo Scientific) and resolved by SDS-PAGE. Molecular weight markers were visualized on gels and immunoblots using a 1:1 mixture of Precision Plus Protein Dual Color and WesternC Standards (BioRad). Proteins were transferred to low fluorescence PVDF membranes (BioRad) using the Trans-Blot Turbo Transfer System (BioRad). Membranes were blocked in 5% nonfat dry milk (BioRad) in TBST (20 mM Tris pH 7.5, 500 mM NaCl, 0.5% Tween 20) for 1 hour, then incubated overnight at 4°C with primary antibodies (see Key Resource Table). After three TBST washes, membranes were incubated with HRP-conjugated secondary antibodies for 1 hour at room temperature. For molecular weight marker visualization in the chemiluminescent channel, Precision Protein StrepTactin-HRP Conjugate (BioRad) was included. Blots were washed and developed with chemiluminescent substrates (GE Amersham ECL, BioRad Clarity Max, or LI-COR WesternSure PREMIUM) and imaged using HyBlot CL film, BioRad ChemiDoc MP, or LI-COR Odyssey XF systems.

Polysome profiling

U937 LARP1 KO and U937 CRISPR control cells were treated with cycloheximide (Thermo Scientific Chemicals) at 100 μg/ml for 30 minutes prior to cells being harvested. Cell pellets were

resuspended in 450 μl of hypotonic lysis buffer (5 mM Tris 7.4 pH, 2.5 mM $MgCl_2$, 1.5 mM KCl, 0.5% Triton-X 100, 0.5% SDS, 300μg/ml cycloheximide, 3 mM DTT, 240 U/ml Protector RNase Inhibitor, 10 μL/ml Protease Inhibitor Cocktail Set III) and tumbled at 4°C for 5 minutes. Cell debris was separated by 20 minutes centrifugation at 21000 rcf, and OD at 254 nm was measured. 400 OD of the lysate was layered on top of 5-50% sucrose gradient (20 mM HEPES, 5 mM $MgCl_2$, 100 mM KCl, 5-50% sucrose, 100 U/ml heparin, 100 μg/ml cycloheximide, 1 mM DTT, 20 U/ml Protector RNase Inhibitor, 5μL/ml Protease Inhibitor Cocktail Set V) prepared on Gradient Master 108 (Biocomp), and centrifuged for 2 hours at 35000 RPM, 4°C (Beckman Coulter Optima XPN-80 Ultracentrifuge, SW 41 Ti rotor). Polysome profiles were obtained by running the sucrose gradient at constant rate 1.5 ml/min (BRANDEL syringe pump SYN-202), reading absorption of 254 nm wavelength (BRANDEL type 11 optical unit and UA-6 absorbance detector). Polysomal samples were obtained by pooling corresponding fractions collected during the run (BRANDEL R1 fraction collector) and subjected to RNA extraction (Qiagen AllPrep RNA/Protein Kit #80404) in parallel with saved sample of input lysate.

Seahorse mitochondrial function analysis

Sample preparation and data acquisition:

Mitochondrial respiration and glycolytic function were evaluated using an Agilent Seahorse XF extracellular flux analyzer and the XF Cell Mito Stress Test kit (Agilent 03015-100), following the manufacturer's protocol with minor adaptations. Seahorse sensor cartridges were hydrated overnight in XF calibrant at 37°C in a non-CO_2 incubator. Seahorse XF cell culture plates were coated with Cell-Tak (Corning 354240; 22.3 μg/mL in 0.1 M $NaHCO_3$, pH 8.0; 20 μL/well) for at least 20 min at room temperature, washed twice with molecular grade water, and air-dried. U937 clone 2 sgNT control and U937 clone 2 sg*LARP1* cells were resuspended in Seahorse XF base medium without phenol red (Agilent 103335-100) supplemented with 2 mM glutamine, and $1 \cdot 10^5$ cells in 150 μL of assay medium were dispensed per well onto the coated plates. Plates were

centrifuged at 400 · g for 2 min with the brake off to promote uniform adherence and then incubated at 37°C in a non-CO_2 incubator before measurements. Background wells contained assay medium alone. Glucose, oligomycin, FCCP, and rotenone/antimycin A together with 2- deoxyglucose were prepared in assay medium and loaded into the injection ports to achieve final concentrations of 11 mM glucose, 2 μM oligomycin, 0.35 μM FCCP, and 1 μM rotenone/antimycin A plus 25 mM 2-deoxyglucose (Rot/AA/2DG). Oxygen consumption rate (OCR) and extracellular acidification rate (ECAR) were recorded at baseline and after each successive injection.

Data analysis:

Raw OCR and ECAR data were exported and processed as follows. For each well, three consecutive OCR measurements acquired after glucose addition (measurements 4-6) were averaged, and three OCR measurements obtained after Rot/AA/2DG addition (measurements 13-15) were similarly averaged. Basal mitochondrial OCR was defined for each well as the post-glucose average minus the post Rot/AA/2DG average, thereby correcting for non-mitochondrial respiration. ATP-linked respiration was determined by subtracting the OCR following oligomycin treatment (measurement 7) from the post-glucose OCR (measurement 6) for each well. Maximal respiration was defined as the highest OCR value observed after FCCP injection (measurement 10). For ECAR, glucose-stimulated glycolysis was calculated as the increase in ECAR after glucose injection relative to the pre-glucose baseline (measurement 4 minus measurement 3), and maximal glycolytic capacity was taken as the highest ECAR value observed after glucose addition (measurement 7). Resulting values were used for graphical representation and statistical analysis.

RNA sequencing

Sample preparation:

Frozen RNA samples obtained from polysome profiling experiments were sent to Novogene Corporation for processing. RNA quantity, integrity, and purity were assessed using the Agilent 5400 Fragment Analyzer System. Samples with an RNA Integrity Number (RIN) above 6.0 qualified for library preparation. Messenger RNA (mRNA) was purified from total RNA using poly-T oligo-attached magnetic beads. Following fragmentation, first-strand cDNA synthesis was performed using random hexamer primers, followed by second-strand cDNA synthesis. Library preparation included end repair, A-tailing, adapter ligation, size selection, amplification, and purification. The final libraries were assessed using a Qubit fluorometer and real-time PCR for quantification, as well as a Bioanalyzer for size distribution analysis. Quantified libraries were pooled and sequenced on Illumina platforms based on the effective library concentration and desired data output.

Data acquisition:

Prepared RNA libraries were sequenced on the Illumina NovaSeq X Plus platform with 25B flowcell, using 150 bp paired-end reads with a read depth of 20 million reads per sample. The resulting raw data were assessed for the distribution of sequencing quality scores, sequencing error rates, and GC content. Subsequently, the raw sequencing reads underwent filtering to remove those containing adapter sequences, reads with more than 10% undetermined bases (N), and reads in which over 50% of the bases had low quality (Q score ≤ 5). The sequences of the library adapters used were as follows: 5’ adapter (5’-AATGATACGGCGACCACCGAGATCTACAC(i5Index)ACACTCTTTCCCTACACGACGCTCTTCCGATCT-3’) and 3’ adapter (5’-CAAGCAGAAGACGGCATACGAGAT(Reverse complementary sequence of i7Index)GTGACTGGAGTTCAGACGTGTGCTCTTCCGATC-3’). Following preliminary processing, the RNA sequencing data were subjected to further analysis in-house at Northwestern University.

Data analysis:

All FASTQ files were processed using the nf-core/rnaseq pipeline (v3.14.0) (1) implemented in Nextflow with the Northwestern University Quest HPC genomic node configuration (nextflow run nf-core/rnaseq -profile nu_genomics --genome GRCm38). Lane-level reads were trimmed using Trim Galore! (v0.6.5), aligned to the GRCm38 reference genome with STAR, and quantified using Salmon. Differential expression analysis was performed in R (v4.3.3) using DESeq2 (v1.42.1) (2), with drug treatment or genotype used as the explanatory variable. Principal component analysis (PCA) confirmed within-group variation, and no outliers were detected.

Global proteomics

Sample preparation:

Quantified protein lysates (100µg) were submitted to the Proteomics core in 8M urea lysis buffer containing phosphatase and protease inhibitors. Proteins were reduced with dithiothreitol (5mM final concentration), alkylated with iodoacetamide (15mM final concentration) then quenched with dithiothreitol (15mM). Proteins were initially digested using Lys-C enzyme (1µg, Promega) for 4 hours, then 100mM ammonium bicarbonate was added to dilute the urea concentration below 2M. Trypsin enzyme (1µg, Promega) was added, and digestion was performed overnight at 37ºC on a Thermomixer at 500rpm. The digest was halted by acidification and peptides were desalted by C18 solid phase extraction (Waters SepPak). Eluted peptides were dried by vacuum centrifugation then resuspended in 20µl of 0.1% aqueous formic acid, shaken and sonicated for 10 minutes each, then centrifuged for 5 minutes at 10,000 ×g.

Data acquisition:

Peptide supernatants were transferred to glass auto-sampler vials then placed in the auto-sampler of a Thermo Scientific Vanquish Neo nano liquid chromatography instrument. 3µl of sample was loaded onto a C18 trap column (Thermo Acclaim PepMap 100, 75µm × 2cm) then transferred to a C18 analytical column (PepMap Neo, 75µm × 25cm). Peptides were separated

using an increasing gradient of organic mobile phase (80% acetonitrile and 0.1% aqueous formic acid) and nano-electrosprayed into the heated source of a Thermo Scientific Orbitrap Exploris 480 mass spectrometer. A 120-minute method acquired raw data files with the following settings. Full scan (MS1): 2 second cycle time, 120000 resolution, 350-1600 m/z mass range, AGC target standard and auto maximum injection time mode. Settings for ddMS2 (MS/MS) were: charge states 2+ to 5+ inclusive, 30 second dynamic exclusion +/- 10 ppm, isolation width 1.4 m/z, higher-energy collisional dissociation (HCD) energy 30%, 15000 resolution, AGC target standard and auto maximum injection time mode.

Data analysis:

Raw mass spectrometry data were processed using MaxQuant software (version 2.5.2.0) against the human SwissProt proteome (downloaded on September 30, 2024). Enzyme specificity was set to trypsin, allowing for a maximum of two missed cleavages. Carbamidomethylation of cysteine was defined as a fixed modification, while acetylation of protein N-termini, oxidation of methionine, and deamidation of asparagine and glutamine were specified as variable modifications, with a maximum of 2 variable modifications allowed. LFQ quantification was enabled with a minimum ratio of peptides required for the process set to 2. The resulting protein group file was further analyzed using Perseus software (version 2.0.11). Contaminants, reversed sequences, and entries identified by site were removed from the dataset. LFQ intensities were log2-transformed, and proteins were filtered to retain only those detected in at least 50% of the replicates in at least 1 group (i.e., one sample for the control group or two samples for the LARP1 group). Missing values were imputed using a normal distribution. Data normalization was performed by median subtraction per column. To identify significantly different proteins between conditions, a Student's t-test was applied, and p-values and $\log_2$ fold change differences were computed.

Metabolomics

Sample preparation

sgNT CTRL cells were collected 4 hrs after the various drug treatments. sgNT CTRL and sgLARP1 KO cells were collected ~ 24 hrs after plating under normal growth conditions. Cells were washed with sodium chloride solution then resuspended in 80% methanol for multiple freeze-thaw cycles at −80°C and stored at −80°C overnight to precipitate the hydrophilic metabolites. Samples were centrifuged at 20,000 · *g* and the supernatant in methanol was removed for metabolite analysis. Extraction solution was dried using SpeedVac. 60% acetonitrile was added to the tube for reconstitution following by overtaxing for 30 sec. Samples solution was then centrifuged for 30 min @ 20,000 g, 4 °C. Supernatant was collected for LCMS analysis. The remaining protein pellets were dissolved with 8 mol/L urea and total protein was quantified using BCA assay. Total protein amount was used for equivalent loading for high performance liquid chromatography and high-resolution mass spectrometry and tandem mass spectrometry (HPLC-MS/MS) analysis.

Data acquisition:

Samples were analyzed by High-Performance Liquid Chromatography and High-Resolution Mass Spectrometry and Tandem Mass Spectrometry (HPLC-MS/MS) as previously described (3). Specifically, system consisted of a Thermo Q-Exactive in line with an electrospray source and an Ultimate3000 (Thermo) series HPLC consisting of a binary pump, degasser, and auto-sampler outfitted with a Xbridge Amide column (Waters; dimensions of 3.0 mm × 100 mm and a 3.5 µm particle size). The mobile phase A contained 95% (vol/vol) water, 5% (vol/vol) acetonitrile, 10 mM ammonium hydroxide, 10 mM ammonium acetate, pH = 9.0; B was 100% Acetonitrile. The gradient was as following: 0 min, 15% A; 2.5 min, 64% A; 12.4 min, 40% A; 12.5 min, 30% A; 12.5-14 min, 30% A; 14-21 min, 15% A with a flow rate of 150 µl/min. The capillary of the ESI source

was set to 275 °C, with sheath gas at 35 arbitrary units, auxiliary gas at 5 arbitrary units and the spray voltage at 4.0 kV. In positive/negative polarity switching mode, an *m/z* scan range from 60 to 900 was chosen and MS1 data was collected at a resolution of 70,000. The automatic gain control (AGC) target was set at 1×10^6 and the maximum injection time was 200 ms. The targeted ions were subsequently fragmented, using the higher energy collisional dissociation (HCD) cell set to 30% normalized collision energy in MS2 at a resolution power of 17,500. Besides matching m/z, target metabolites are identified by matching either retention time with analytical standards and/or MS2 fragmentation pattern. Data acquisition and analysis were carried out by Xcalibur 4.1 software and Tracefinder 4.1 software, respectively (both from Thermo Fisher Scientific).

Data analysis:

Metabolites that were below detection in all samples were removed from analysis. After identification, samples were normalized by taking the peak AUC for each metabolite per sample and dividing by the quotient of the total ion count (TIC) per sample over the lowest TIC in the batch. Data was analyzed using Metaboanalyst 6.0. Subsequent transformation of normalized data was carried out with auto scaling to account for heteroscedasticity and a 2-fold change and raw *p* value of 0.1 was used as a cutoff for significance (4).

Gene annotation and protein function enrichment analysis

Gene name lists from both Proteomics and RNA-seq were submitted to the Metascape database, a gene annotation and analysis resource (http://metascape.org/), for pathway and process enrichment analysis (5, 6). For each given gene list, Metascape carries pathway and process enrichment analysis using the following ontology sources: GO Biological Processes, KEGG Pathway and Reactome Gene Sets. All genes in the genome were used as the enrichment background. Terms with p-value < 0.01, minimum count 3, and enrichment factor > 1.5 are collected and grouped into clusters based on their membership similarities. More specifically, p-values are calculated based on accumulative hypergeometric distribution, q-values are calculated

using the Banjamini-Hochberg procedure to account for multiple testing. Kappa scores were used as the similarity metric when performing hierarchical clustering on the enriched terms and then sub-trees with similarity > 0.3 are considered a cluster. The most statistically significant term within a cluster is chosen as the one representing the cluster.

Multi-omics Factor Analysis (MOFA)

Multi-omics data integration was performed using Multi-Omics Factor Analysis (MOFA), an unsupervised latent factor modeling approach that identifies shared and modality-specific sources of variability across multiple omics layers. Input (total RNA) and polysome-associated RNA-seq data, proteomics, and metabolomics data were included in the analysis. RNA-seq count data were normalized using the trimmed mean of M-values (TMM) method and transformed to log counts per million (logCPM). To reduce noise and computational burden, the 5,000 most variable genes were selected from the input RNA-seq dataset and used consistently for both input and polysomal RNA-seq views. Proteomics data were also filtered to retain the most variable features, while all metabolomics features were included.

MOFA models were trained using four latent factors, selected based on sample size, convergence diagnostics, and interpretability. The fraction of variance explained by each factor within each omics layer was calculated, and factor values were tested for association with experimental covariates, including genotype and drug treatment, using linear models with multiple-testing correction.

To characterize transcriptional and translational programs associated with latent factors, feature weights from the polysomal RNA-seq view were examined. Genes with extreme factor loadings (absolute Z-score ≥ 3) were selected for further visualization. For heatmap analyses, total and polysome-associated RNA-seq expression data were visualized using identical gene and sample ordering, with per-gene Z-score normalization applied within each dataset to enable direct

comparison. Relationships between MOFA factor values and global RNA abundance were assessed by comparing factor values to summed normalized RNA-seq signal across samples.

Comparison of LARP1 expression in AML subgroups

Relative *LARP1* mRNA expression for different AML subgroups was obtained through BloodSpot (https://www.bloodspot.eu) (7) using AML TCGA (8) and BEAT AML (9) datasets and the default settings. Relative protein expression of LARP1 was obtained from proteomics data from the LeyLab AML proteomic database (https://www.leylab.org/amlproteome/) (10) by querying "LFQ- Single protein by key AML fusions."

REFERENCES:

1. Di Tommaso P, Chatzou M, Floden EW, Barja PP, Palumbo E, Notredame C. Nextflow enables reproducible computational workflows. Nat Biotechnol. 2017;35(4):316-9.

2. Love MI, Huber W, Anders S. Moderated estimation of fold change and dispersion for RNA-seq data with DESeq2. Genome Biol. 2014;15(12):550.

3. Villa E, Sahu U, O'Hara BP, Ali ES, Helmin KA, Asara JM, et al. mTORC1 stimulates cell growth through SAM synthesis and m(6)A mRNA-dependent control of protein synthesis. Mol Cell. 2021;81(10):2076-93 e9.

4. Riesenberg BP, Hunt EG, Tennant MD, Hurst KE, Andrews AM, Leddy LR, et al. Stress-Mediated Attenuation of Translation Undermines T-cell Activity in Cancer. Cancer Res. 2022;82(23):4386-99.

5. Soonthornvacharin S, Rodriguez-Frandsen A, Zhou Y, Galvez F, Huffmaster NJ, Tripathi S, et al. Systems-based analysis of RIG-I-dependent signalling identifies KHSRP as an inhibitor of RIG-I receptor activation. Nat Microbiol. 2017;2:17022.

6. Tripathi S, Pohl MO, Zhou Y, Rodriguez-Frandsen A, Wang G, Stein DA, et al. Meta- and Orthogonal Integration of Influenza "OMICs" Data Defines a Role for UBR4 in Virus Budding. Cell Host Microbe. 2015;18(6):723-35.

7. Gislason MH, Demircan GS, Prachar M, Furtwangler B, Schwaller J, Schoof EM, et al. BloodSpot 3.0: a database of gene and protein expression data in normal and malignant haematopoiesis. Nucleic Acids Res. 2024;52(D1):D1138-D42.

8. Cancer Genome Atlas Research N, Ley TJ, Miller C, Ding L, Raphael BJ, Mungall AJ, et al. Genomic and epigenomic landscapes of adult de novo acute myeloid leukemia. N Engl J Med. 2013;368(22):2059-74.

9. Tyner JW, Tognon CE, Bottomly D, Wilmot B, Kurtz SE, Savage SL, et al. Functional genomic landscape of acute myeloid leukaemia. Nature. 2018;562(7728):526-31.

10. Kramer MH, Zhang Q, Sprung R, Day RB, Erdmann-Gilmore P, Li Y, et al. Proteomic and phosphoproteomic landscapes of acute myeloid leukemia. Blood. 2022;140(13):1533-48.
